# OTUD5 maintains STAT1/2 stability and promotes IFNγ-driven intestinal inflammation

**DOI:** 10.1016/j.jbc.2026.113420

**Published:** 2026-08-13

**Authors:** Huiyuan Guan, Changzhou Cai, Jiewei Wang, Zhaoxue Liu, Jin Peng, Xupeng Bai, Lingzhi Wu, Kun Jiang, Xinghua Zhen, Chaohui Yu, Pumin Zhang, Zhe Shen

**Affiliations:** 1 Zhejiang Provincial Key Laboratory of Pancreatic Disease, The First Affiliated Hospital of Zhejiang University School of Medicine, Hangzhou, Zhejiang, China; 2 Institute of Translational Medicine, Zhejiang University Medical School, Hangzhou, Zhejiang, China; 3 Department of Gastroenterology, The First Affiliated Hospital of Zhejiang University School of Medicine, Hangzhou, Zhejiang, China; 4 Chaser Therapeutics Inc., Hangzhou, Zhejiang, China; 5 Cancer Center, Zhejiang University, Hangzhou, Zhejiang, China

**Keywords:** anti-TNFα resistance, IFNγ-ISGF3 signaling, IBD, OTUD5, STAT1/2

## Abstract

Inflammatory bowel disease is characterized by unresolved mucosal inflammation driven mainly by TNFα and/or interferon gamma (IFNγ). While anti-TNFα biologics are a clinical mainstay, therapeutic resistance remains a significant hurdle. The molecular mechanisms that sustain inflammation in anti-TNFα nonresponders are not fully understood. In human colon biopsies, we identified significant upregulation of the deubiquitinase OTUD5 in nonresponders compared to responders, suggesting its involvement in therapy resistance. Using an intestinal epithelial cell -specific *Otud5* KO mouse model, we show that *Otud5* deficiency significantly alleviated the IFNγ-dependent colitis induced by dextran sulfate sodium, as evidenced by reduced weight loss and diminished infiltration of Ly6C^+^ inflammatory monocytes. Mechanistically, IFNγ induces OTUD5 expression through a nontranscriptional mechanism; in turn, OTUD5 stabilizes STAT1 and STAT2 by preventing their ubiquitination and subsequent degradation. This sustains IFNγ-ISGF3 signaling, which directly drives the expression of CCL8, a critical chemokine for monocyte recruitment. Targeting this pathway with a newly identified small-molecule inhibitor, CT1170, which exhibits potent activity against OTUD5, blocked the IFNγ-ISGF3-CCL8 axis, halted colitis progression, and suppressed colitis-elicited tumorigenesis. These findings were validated in human inflammatory bowel disease organoids, where CT1170 effectively disrupted IFNγ-driven inflammatory signaling.

Inflammatory bowel disease (IBD) is a global health issue, the incidence of which has been gradually increasing worldwide (1, 2, 3). The condition includes ulcerative colitis (UC) and Crohn’s disease (CD). UC is characterized by relapsing and remitting mucosal inflammation, starting in the rectum and extending to proximal segments of the colon (4, 5), whereas CD is characterized by segmental, asymmetrical, and transmural inflammation. CD can affect any segments of the gastrointestinal tract (the most common being the terminal ileum and colon) (6, 7). The pathophysiology of IBD is complex, involving multiple factors both environmental and genetical. It is believed that IBD starts with environmental exposures that result in the disruption of the intestinal barrier and subsequent invasion by gut microbes, triggering innate immune responses first in gut resident immune cells as well as the intestinal epithelial cells (IECs) and then the adaptive immune responses (8). The responses include the production of a large number of cytokines/chemokines such as tumor necrosis factor alpha (TNFα), interferons, and interleukins (9). If the immune responses are not resolved properly, inflammation and colitis ensue, resulting in intestinal ulcers that often require clinical attention.

Current treatment options for IBD include 5-a*minosalicylic acids*, corticosteroids, immunomodulators, and biologics (8), with the intention to suppress inflammation. One of the biologics, the anti-TNFα antibodies, such as infliximab (IFX), has been a cornerstone in the management of IBD, including both CD and ulcerative colitis (10, 11, 12, 13). The therapy targets the pro-inflammatory cytokine TNFα to reduce inflammation and induce remission in IBD patients. However, a substantial proportion of patients either do not respond to the therapy initially (primary nonresponders (NR)) or lose response over time (secondary NRs) (14, 15, 16, 17). For example, it was reported that up to 40% of patients were primary NRs in one study (18). The reasons for nonresponse or loss of response to anti-TNF therapy are multifactorial. Immunogenicity, referring to the development of anti-drug antibodies, is a significant factor contributing to the loss of efficacy (16), but many other factors including the presence of other inflammatory pathways not targeted by TNFα inhibitors also play a role in the NRs (19). Identifying and targeting those other inflammatory pathways are being actively researched in the efforts to develop alternative agents and strategies to bring therapeutic benefits to those IBD patients nonresponsive to anti-TNFα treatments.

Here we show that the deubiquitinase OTUD5 is significantly upregulated in anti-TNFα NRs and is a critical driver of intestinal inflammation. Genetic deletion of *Otud5* in IEC in mice rendered these animals refractory to dextran sulfate sodium (DSS)-induced colitis as evidenced by reduced weight loss and diminished infiltration of Ly6C^+^ inflammatory monocytes. Mechanistically, we discovered that OTUD5 stabilizes STAT1/2 protein by preventing their ubiquitination and degradation. High levels of OTUD5 sustain the IFNγ-STAT1/2-ISGF3 signaling axis, which directly induces the expression of the chemokine CCL8, a critical driver of monocyte recruitment. We demonstrate that targeting this cascade with a newly identified small-molecule compound, CT1170, which possesses potent activity against OTUD5, suppresses the STAT1/2-CCL8 signaling cascade and halts colitis progression. Importantly, these findings could be recapitulated in human IBD organoids. Furthermore, CT1170 treatment effectively ameliorated colitis-associated tumorigenesis in an azoxymethane (AOM)/DSS model. In short, our findings suggest that the OTUD5-STAT1/2-CCL8 axis represents an important contributing mechanism to anti-TNFα resistance and provide strong evidence for OTUD5 inhibition as a potential salvage therapy for anti-TNFα refractory IBD.

## Results

### OTUD5 is up-regulated in IBD patients nonresponsive to anti-TNFα therapy and required for intestinal inflammation

**Figure 1:**
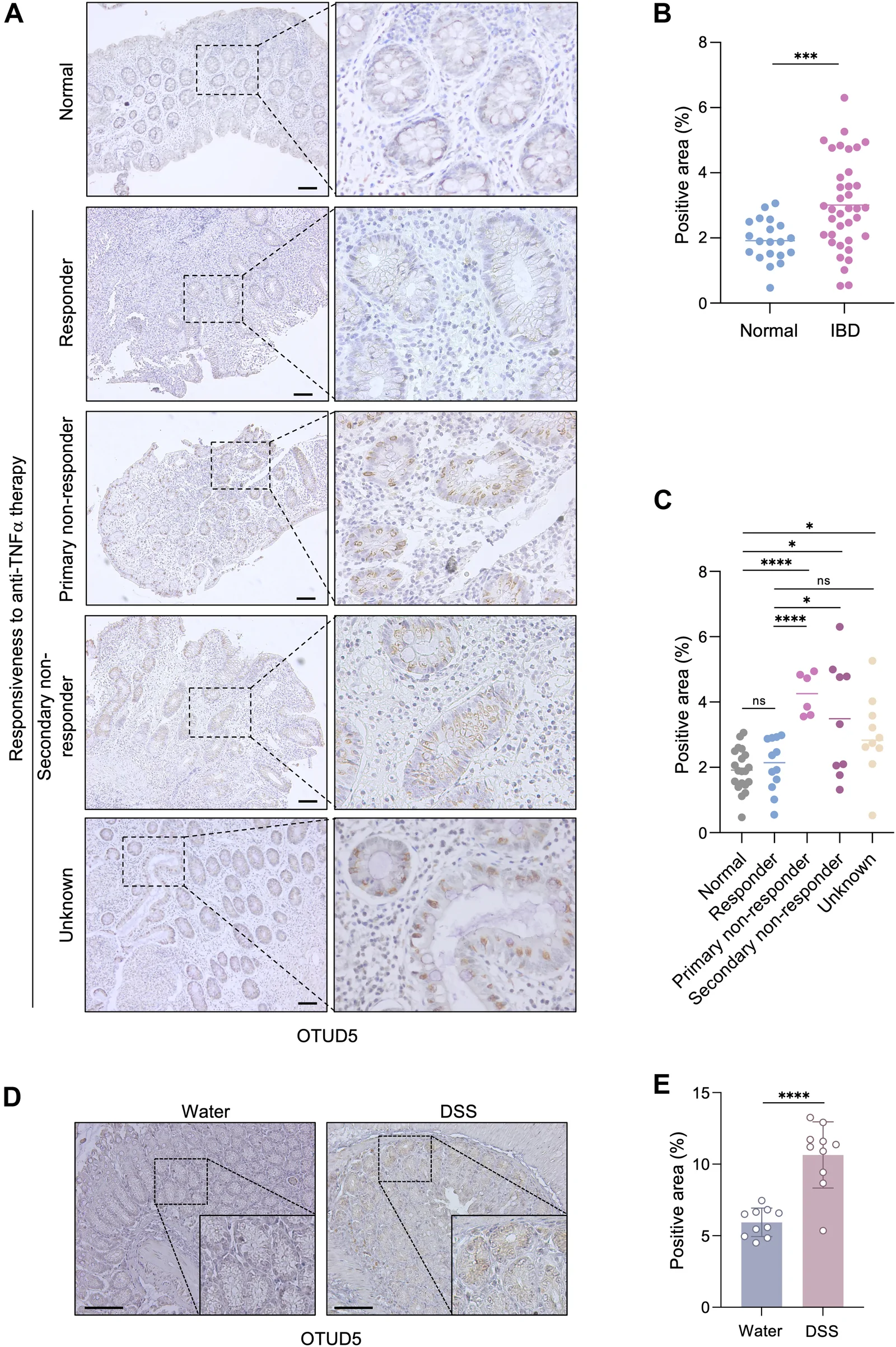
**OTUD5 is up-regulated in IBD patients nonresponsive to anti-TNFα therapy.***A*, IHC staining of OTUD5 in the colon biopsy including 20 normal colon tissue samples (from the excision of intestinal polyps) and 37 tissue samples from IBD patients divided to three groups according to their responsiveness to anti-TNFα therapy: responders (n = 12), primary NRs (n = 6), secondary NRs (n = 9), and unknown (n = 10). Scale bar: 100 μm. *B and C,* quantitation of *(A)*. *D,* (IHC staining of OTUD5 in colon tissues from the mice treated with Water (n = 10) or DSS (n = 10) for 3 days. Scale bar: 100 μm. *E,* quantitation of *(D)*. Data are presented as mean ± SD. ∗, *p* < 0.05; ∗∗, *p* < 0.01; ∗∗∗, *p* < 0.001; ∗∗∗∗, *p* < 0.0001. DSS, dextran sulfate sodium.

As reported previously (20), we also found that OTUD5 was up-regulated in colon biopsies from IBD patients (Fig. 1, *A* and *B*). However, stratifying the samples by responses to anti-TNFα therapy demonstrated a significant upregulation of OTUD5 in the therapy NRs than in the responders (Fig. 1, *A* and *C*). In the DSS (dextran sulfate sodium)-induced murine model of intestinal inflammation, OTUD5 is also highly upregulated in the IECs (Fig. 1, *D* and *E*). Interestingly, it was reported previously that DSS could still induce intestinal inflammation and tissue damage in TNFα KO mice (21), which indicates that the DSS-induced IBD-like conditions in mice are independent of TNFα signaling, making the DSS model a surrogate for anti-TNFα therapy resistance in human IBD patients. We therefore reasoned that *OTUD5* might participate in a process of intestinal inflammation and IBD pathology which is distinct from TNFα-mediated process and might be closely associated with the anti-TNFα therapy resistance seen in a portion of IBD patients.

**Figure 2:**
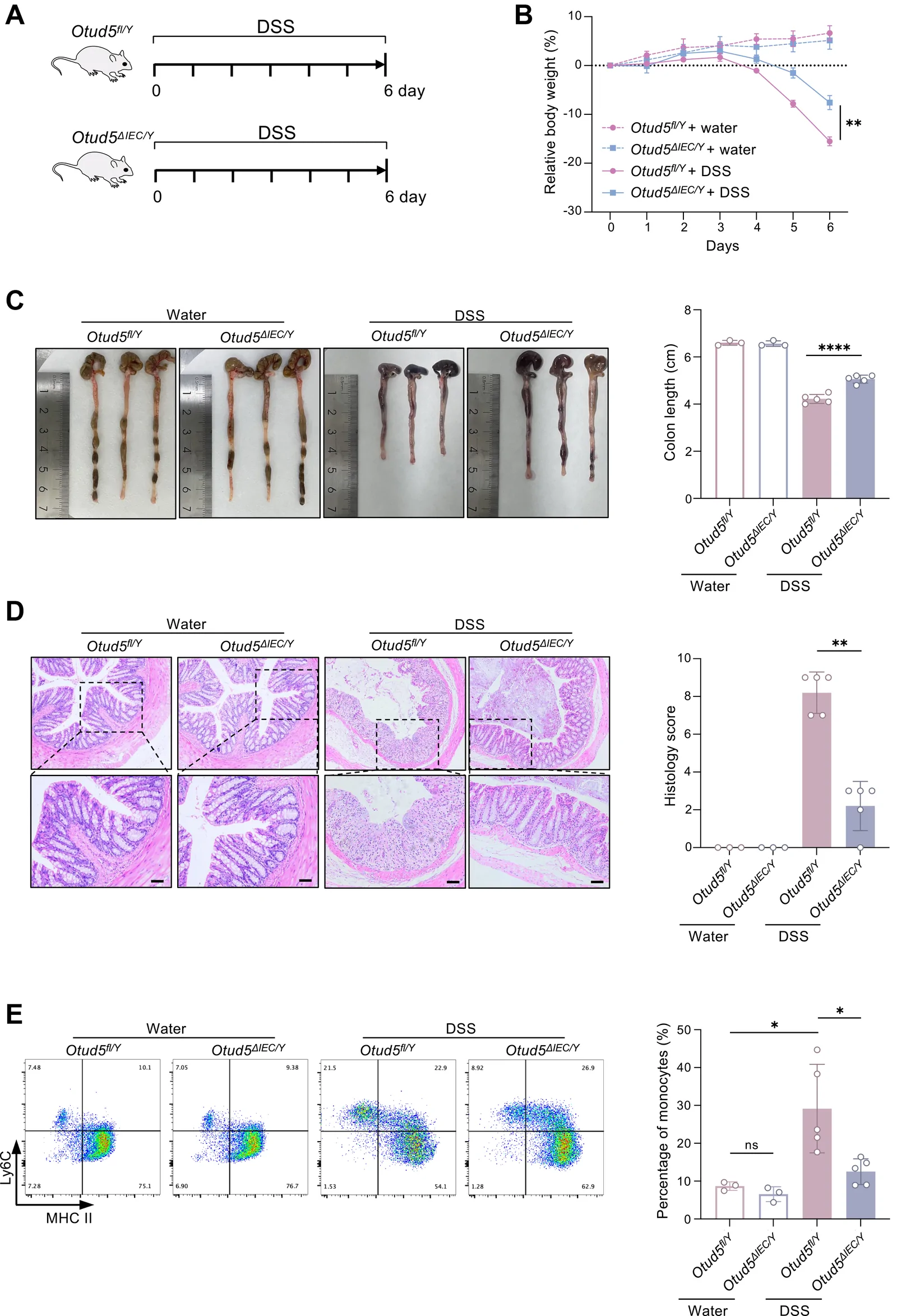
**Deletion of *Otud5* in IECs alleviates DSS-induced colitis.***A,* schematic diagram of DSS-induced colitis model (n = 3 per group for water controls and n = 5 per group for DSS-treated mice). *B*, body weight measurements of the mice during DSS treatment. *C,* the colons were collected on day 6 of DSS treatment and their lengths were measured. *D,* colon tissues from *(C*) were H&E stained and scored. Scale bar: 100 μm. *E,* flow cytometric analysis of Ly6C^+^MHC II^-^ inflammatory monocytes in the LP. Representative flow results from 6-days water- or DSS-treated mice are shown. Data are presented as mean ± SD (except in *B,* mean ± S.E.M. was presented). ∗, *p* < 0.05; ∗∗, *p* < 0.01; ∗∗∗, *p* < 0.001; ∗∗∗∗, *p* < 0.0001. DSS, dextran sulfate sodium.

To determine the function of the DUB in intestinal inflammation, we generated mice with IEC-specific KO of *Otud5* (*Otud5*^*ΔIEC/Y*^, the gene is located on X chromosome) by bringing a floxed *Otud5* allele (22) together with a *Vil1-Cre* transgene and subjected these mice (along with *Otud5*^*fl/Y*^ control mice) to DSS treatment for 6 days (Fig. 2*A*). As shown in Figure 2*B*, the deletion of *Otud5* resulted in much less weight loss caused by the DSS treatment. The colons from *Otud5*^*ΔIEC/Y*^ mice were much longer than that from control mice (Fig. 2*C*) and histological examination of the intestine showed much less tissue damage in the *Otud5*^*ΔIEC/Y*^ animals than in controls (Fig. 2*D*). The infiltration of Ly6C^+^ MHC II^-^ inflammatory monocytes (assayed through stepwise gating of the immune cells in the lamina propria (LP), Fig. S2), the driving force in IBD progression (23, 24, 25), also decreased in *Otud5*^*ΔIEC/Y*^ mice (Fig. 2*E*). These results all indicate that *Otud5* is critical for the development of inflammatory conditions caused by DSS in the intestine.

### *Otud5* in IECs promotes the infiltration of inflammatory monocytes infiltration *via* CCL8

**Figure 3:**
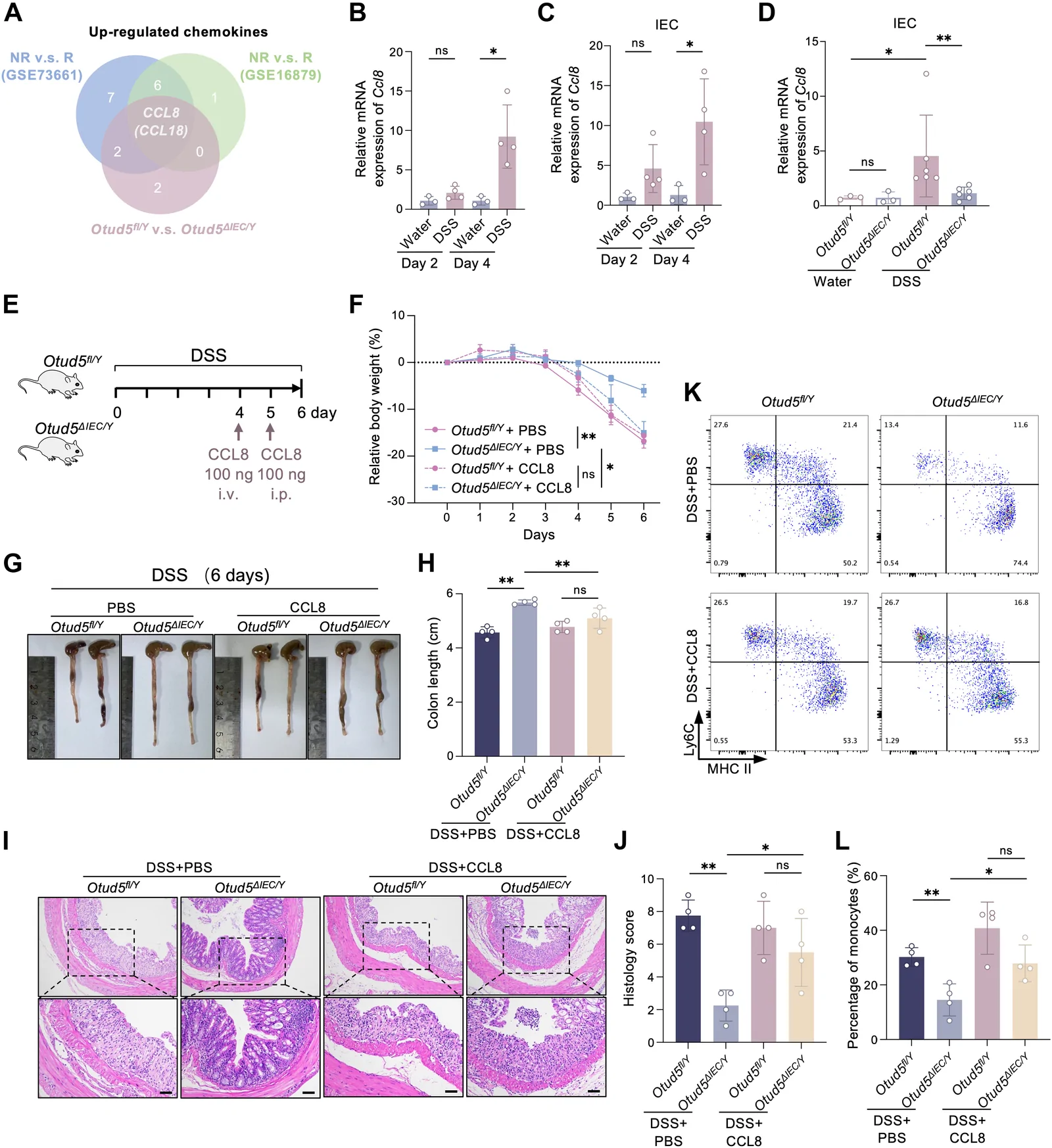
**OTUD5 in IECs sustains inflammatory monocytes infiltration and intestinal inflammation *via* CCL8.***A,* Venn diagram identifying the overlap of significantly upregulated chemokines across three comparative groups. The *blue* circle denotes chemokines that are highly expressed in NR group compared to responder (R) group, with data derived from GSE73661. The *green* circle denotes chemokines that are highly expressed in NR group compared to responder (R) group, with data derived from GSE16879. The lavender circle denotes chemokines that are highly expressed in IECs from *Otud5*^*fllY*^ mice (n = 3) compared to *Otud5*^*ΔIEC/Y*^ mice (n = 3) after 4 days of DSS administration. *B,* qPCR analysis of *Ccl8* expression in colon tissue from the mice treated with water or DSS for two or 4 days (n = 3 for the water group and n = 4 for the DSS group at each time point). *C,* qPCR analysis of *Ccl8* expression in IECs from the mice treated with water or DSS for two or 4 days (n = 3 for the water group and n = 4 for the DSS group at each time point). *D,* qPCR analysis of *Ccl8* expression in IECs isolated from 4-days water- or DSS-treated control and *Otud5* KO mice (n = 3 per group for water controls and n = 6 per group for DSS-treated mice). *E,* schematic diagram of CCL8 administration in DSS-induced colitis model (n = 4 per group). *F,* body weight measurements of the mice during the treatment *(E)*. *G and H,* the colons were isolated from the mice in different treatment groups on day 6. The colon lengths were measured and presented in *(H)*. *I and J,* H&E staining *(I)* and scoring *(J)* of the colons in *(G)*. The scale bar represents 100 μm. *K and L,* flow cytometric analysis of Ly6C^+^MHC II^-^ inflammatory monocytes in the LP. Representative flow results *(K)* and quantitation of the Ly6C^+^MHC II^-^ monocytes from all animals are shown *(L)*. Data are presented as mean ± SD (except in *F,* mean ± S.E.M. was presented). ∗, *p* < 0.05; ∗∗, *p* < 0.01; ∗∗∗, *p* < 0.001; ∗∗∗∗, *p* < 0.0001. DSS, dextran sulfate sodium; IEC, intestine epithelial cell; LP, lamina propria.

These observations led us to consider the underlying mechanism by which OTUD5 sustains inflammatory monocytes infiltration and intestinal inflammation. A reasonable hypothesis is that OTUD5 exerts these effects by upregulating the expression of specific chemokines. We therefore carried out RNA-seq analyses of IECs isolated from 4-days DSS-treated *Otud5*^*fl/Y*^ and *Otud5*^*ΔIEC/Y*^ mice and focused on the chemokines upregulated in *Otud5*^*fl/Y*^ mice. There are five such upregulated chemokines (Figs. 3*A* and S3*A*), among which *Ccl8* has been reported to play a critical role in DSS-induced colitis by recruiting inflammatory monocytes to the intestine (26), the very type of cells believed to drive IBD progression (23, 24, 25). What’s more, in two human datasets from the Gene Expression Omnibus database (GSE73661 and GSE16879, both of which encompass patients who underwent treatment with IFX and were stratified into NR and responder groups based on treatment efficacy), the functional equivalent of *Ccl8*, *CCL18* (27), is also up-regulated along with many more cytokines in the NR group compared to the responders (Figs. 3*A* and S3*B*). Indeed, *Ccl8* was upregulated in the intestine (Fig. 3*B*) as well as the IECs (Fig. 3*C*) isolated from DSS-treated mice. More importantly, this upregulation diminished in the IECs from *Otud5*^*ΔIEC/Y*^ mice (Fig. 3*D*). Taken together, these results point toward *Ccl8* as a potential key mediator in *Otud5-*dependent intestinal inflammation.

To further determine whether *Otud5* promotes monocyte infiltration and intestinal inflammation primarily through *Ccl8*, we exogenously replenished CCL8 *via* intravenous injection followed by intraperitoneal injection in *Otud5*^*ΔIEC/Y*^ and control mice under DSS treatment (Fig. 3*E*). Remarkably, exogenous CCL8 abrogated the anti-inflammatory effects of *Otud5-*deficiency, as evidenced by increased weight loss (Fig. 3*F*), shortened colon length (Fig. 3, *G* and *H*), and deteriorated histological scores (Fig. 3, *I* and *J*). More importantly, exogenous replenishment of CCL8 restored the infiltration of Ly6C^+^ MHC II^-^ inflammatory monocytes in *Otud5*^*ΔIEC/Y*^ mice (Fig. 3, *K* and *L*). Of note, while systemic administration of recombinant CCL8 successfully restores Ly6C^+^ monocyte infiltration in our model, this delivery route creates a global chemokine enrichment rather than a strictly localized, epithelium-derived gradient. Although this systemic replenishment cannot fully recapitulate the precise spatial dynamics of endogenous CCL8 secreted by IECs, it functionally confirms that CCL8 is capable of driving monocyte recruitment downstream of the altered epithelial signaling. These findings collectively suggest that the function of *Otud5* in the infiltration of inflammatory monocytes and in the intestinal inflammation are largely mediated by CCL8.

### STAT2 is a potential transcriptional activator of *Ccl8* and driver of anti-TNFα therapy resistance

**Figure 4:**
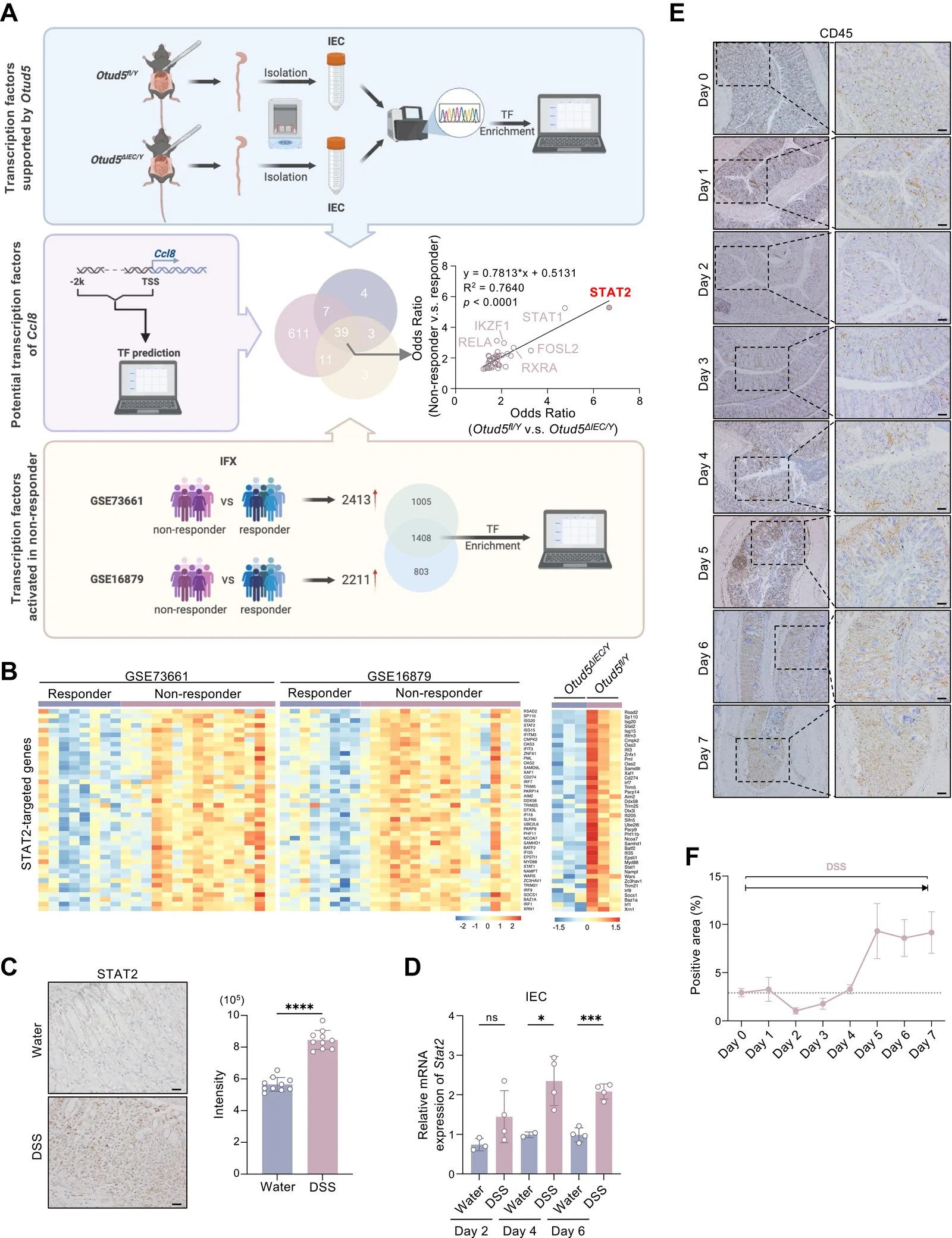
**STAT2 signaling is up-regulated in IBD patients nonresponsive to infliximab.***A,* schematic representation of the workflow for identifying potential TFs of gene *Ccl8*. *(Top panel)* IECs from *Otud5*^*fl/Y*^ or *Otud5*^*ΔIEC/Y*^ mice were collected after DSS treatment (for 4 days), followed by RNA sequencing and TF enrichment analysis. *(Bottom panel)* an intersection of upregulated genes (NR group *versus* responder group) from two datasets (GSE73661 and GSE 16879) yielded 1408 common upregulated genes, followed by TF enrichment analysis. *(Medial-left panel)* The 2 kb sequence upstream of the transcription start site of *Ccl8* was utilized for TF prediction. *(Center panel)* venn diagram identifying the overlap of TFs described above. *(Medial-right panel) scatter plot* and linear regression analysis of shared TFs. X-axis and Y-axis represent Odds Ratio values from the *top* and *bottom* panels, respectively.*B,* heatmap of the STAT2-targeted genes derived from the *top* and *bottom panel* datasets in *(A)*. *C*, IHC staining of STAT2 in colon tissue from mice treated with Water (n = 10) or DSS (n = 10) for 6 days. The scale bar represents 20 μm. *D,* qPCR analysis of the relative mRNA expression levels of *Stat2* in IECs from the mice treated with DSS for indicated days. Water group: n = 3 *(Day 2)*, n = 2 *(Day 4)* and n = 4 *(Day 6)*. DSS group: n = 4 for each group at each time point. *E,* IHC staining of CD45 in colon tissue from mice treated with DSS for indicated days. The scale bar represents 50 μm. *F,* quantitation of CD45 expression in *(E)*. Data are presented as mean ± SD. ∗, *p* < 0.05; ∗∗, *p* < 0.01; ∗∗∗, *p* < 0.001; ∗∗∗∗, *p* < 0.0001. DSS, dextran sulfate sodium; IEC, intestine epithelial cell; IHC, immunohistochemistry; LP, lamina propria; NR, non-responder; TF, transcription factor.

Next, we sought to investigate the mechanism by which *Otud5* regulates *Ccl8* expression. Given the fact that OTUD5 is a deubiquitinase, we proposed that it regulates a transcription factor(s) (TF) which in turn activates *Ccl8* expression and this TF(s) likely also underlie the anti-TNFα therapy resistance in IBD patients. We first performed TF prediction using the AnimalTFDB v4.0 database (28) (https://guolab.wchscu.cn/AnimalTFDB4) to identify (TFs which can bind to the promoter region of *Ccl8* (from −2000 to −1 bp). The analysis identified a total of 668 potential TFs (Fig. 4*A*, left of the mid panel, and Table S1). Next, with the RNA-seq data (Fig. 3*A*), we generated a list of genes down-regulated in the IECs of *Otud5*^*ΔIEC/Y*^ mice treated with DSS and then employed the ChEA3 (29) (ChIP-X Enrichment Analysis 3, https://maayanlab.cloud/chea3) to enrich TFs which may activate the expressions of these genes using the ENCODE CHIP-seq library as the data source (Fig. 4*A*, top panel). A total of 53 TFs were identified through this analysis (Fig. 4*A*, mid panel, and Table S2). Thirdly, we interrogated the datasets GSE73661 and GSE16879 to generate a list of 1408 genes that were significantly upregulated in the NR group in both datasets and then performed similar analyses to associate TFs with this list of genes (Fig. 4*A*, bottom panel). A total of 56 TFs were identified (Fig. 4*A*, mid panel, and Table S2). Among the TFs identified through these three analytic excises, there are 39 common ones (Fig. 4*A*, center of the mid panel). Scatter plotting of these 39 TFs based on their odd ratio (OR) values obtained from *Otud5*^*fl/Y*^ *versus Otud5*^*ΔIEC/Y*^ comparison and from NR *versus* responder comparison (Fig. 4*A*, right of the mid panel) showed a significant correlation between the human and murine datasets, indicating a high degree of consistency between the signals upregulated in NRs and in DSS-treated mice. STAT2 stands out from the plotting as it exhibited the highest OR values in both human and murine datasets, implicating STAT2 as a major TF that drives the expression of genes including *Ccl8* to inflict intestinal inflammation and is responsible for anti-TNFα therapy resistance.

Indeed, STAT2 target genes are upregulated in anti-TNFα therapy resistant patients (Fig. 4*B*), but were not upregulated in the IECs of *Otud5*-deficient mice treated with DSS (Fig. 4*B*), indicating that STAT2 signaling is prominently active in the nonresponsive patients, and requires *Otud5* to be functional. In agreement with that, we observed an upregulation of STAT2 in the IECs of DSS-treated mice (Fig. 4, *C* and *D*), and the upregulation occurred early during DSS-treatment (perhaps as early as the second day, although the statistical significance was not reached until the fourth day) (Fig. 4*D*). Interestingly, the immune cell infiltration did not manifest until day 5 (Fig. 4, *E* and *F*), strongly suggesting that STAT2 plays a priming role in the initiation of DSS-induced colitis pathogenesis. In support of that, *Stat2* was shown previously to be required for DSS-induced colitis in mice (30).

### IFNγ activates *Ccl8* expression *via* the ISGF3 complex in an OTUD5-dependent manner

**Figure 5:**
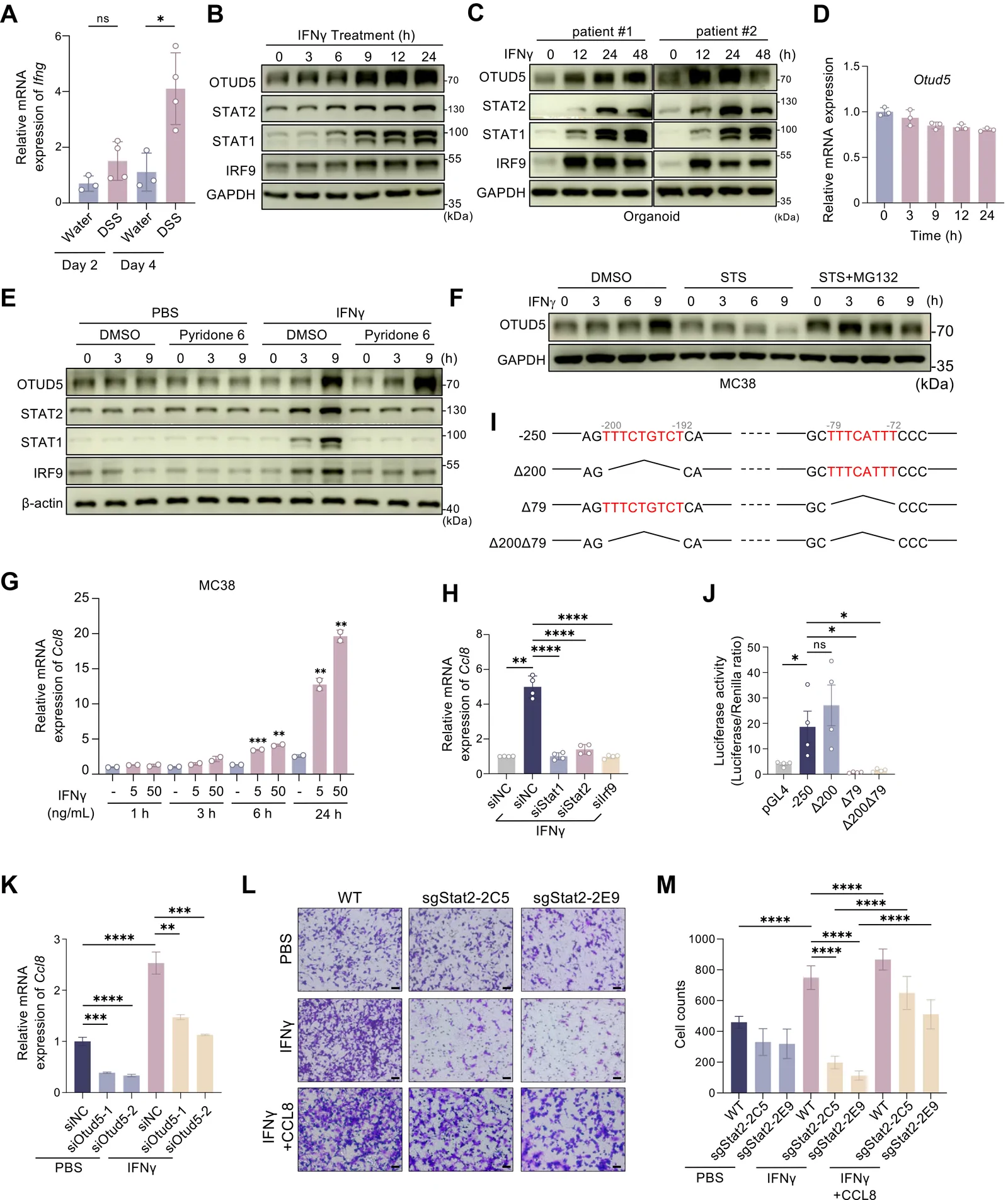
**IFNγ activates Ccl8 expression *via* ISGF3 complex.***A,* qPCR analysis of the relative mRNA expression levels of *Ifng* in the colon tissues from the mice treated with DSS for two or 4 days (n = 3 for the water group and n = 4 for the DSS group at each time point). *B,* Western blot analysis of OTUD5, STAT1, STAT2 and IRF9 in MC38 cells treated with IFNγ (50 ng/ml) for the indicated time periods. *C,* Western blot analysis of OTUD5, STAT1, STAT2 and IRF9 in IBD patients-derived colon organoids treated with IFNγ (50 ng/ml) for the indicated time periods. *D,* qPCR analysis of *Ccl8* expression in MC38 cells treated with IFNγ (50 ng/ml) for the indicated time periods. *E,* Western blot analysis of OTUD5, STAT1, STAT2 and IRF9 in MC38 cells treated with PBS or IFNγ (50 ng/ml) in the presence or absence of pyridone 6 (20 μM, 2 h prior to PBS or IFNγ) for the indicated time periods. *F,* Western blot analysis of OTUD5 expression in MC38 cells treated with IFNγ in the presence or absence of staurosporine (STS, 1 μΜ) over the indicated time periods. The cells in STS + MG132 group were pretreated with MG132 for 2 h prior to STS exposure. *G,* qPCR analysis of *Ccl8* expression in MC38 cells treated with PBS or IFNγ (5 or 50 ng/ml) for the indicated time periods. *H,* qPCR analysis of *Ccl8* expression levels in MC38 cells 48 h after siRNA transfection followed by IFNγ (50 ng/ml) treatment for 6 h. *I,* schematic diagram of the *Ccl8* promoter. Potential ISRE sites were highlighted in *red*. *J,* dual-luciferase reporter assays were performed in MC38 cells 48 h after the transfection of pGL4 plasmids containing the DNA fragments shown in *(H*) followed by IFNγ treatment (50 ng/ml, 6 h). Renilla luciferase activity was used as transfection efficiency control. *K,* qPCR analysis of *Ccl8* expression in MC38 cells depleted of *Otud5* and treated with IFNγ (50 ng/ml) for 4 h. *L,* trans-well chemotaxis assay. WT or *Stat2*-KO MC38 cells in the lower chambers were pre-treated with IFNγ for 6 h followed by overnight coculture with RAW cells in the upper chambers. CCL8 was introduced into selected lower chambers at the start of the coculture period. *M,* quantitation of migrated RAW cells in *(L)*. Data are presented as mean ± SD. ∗, *p* < 0.05; ∗∗, *p* < 0.01; ∗∗∗, *p* < 0.001; ∗∗∗∗, *p* < 0.0001. DSS, dextran sulfate sodium; IFNγ, interferon gamma.

The pattern of *Stat2* expression in the IECss of DSS-treated mice suggests that the damage to the epithelium by DSS somehow activates *Stat2*. It is known that *Stat2* can be activated by type I and type III interferons (31). Accumulating evidence also suggests that STAT2 participates in type II IFNγ-induced up-regulation of specific genes (32, 33, 34). Therefore, we looked into the expression of all three types of interferons in the two datasets (GSE73661 and GSE16879) and found IFNγ (type II interferon) was the only interferon expressed at higher levels in the NR group than in the responder group in the dataset GSE73661(Fig. S4*A*). In GSE16879, the difference did not reach statistical significance, but a higher trend was observed (Fig. S4*A*). Notably, in the DSS-induced colitis model, *Ifng* was up-regulated in the intestine (Fig. 5*A*), coincident with the up-regulation of *Stat2* (Fig. 4*D*). Collectively, these data suggest that IFNγ functions as an upstream activator of *STAT2*, thereby driving intestinal inflammation. Indeed, IFNγ was shown to be necessary for DSS-induced colitis as *Ifng*-deficient mice were refractory to this colitis inducer (35).

IFNγ activates gene expression in a biphasic fashion. In the early phase, IFNγ stimulates the phosphorylation of STAT1 by Janus kinase (JAK) kinases and the phosphorylated STAT1 forms a homodimer (also known as GAF, γ-activated factor) to activate gene expression such as that of *Irf1*. This process requires no new protein synthesis (36, 37). On the other hand, sustained presence of IFNγ activates late phase gene expression in a noncanonical way *via* ISGF3 (Interferon-Stimulated Gene Factor 3) complexes consisting of STAT1, STAT2, and IRF9 (33, 34, 38). The late phase is a slow process that requires new protein synthesis, likely because the ISGF3 components themselves need to be made once their mRNA expression is induced by IFNγ *via* GAF (39). Indeed, IFNγ treatment of MC38 cells (an IEC line derived from colon adenocarcinoma) induced *Stat1*, *Stat2*, and *Irf9* starting at 6 h of the treatment (Fig. S4*B*) and the corresponding proteins became substantially expressed after 9 h of treatment (Fig. 5*B*). Importantly, these inductions could be recapitulated in IBD patients-derived intestinal organoids (Fig. 5*C*). While IFNγ induced OTUD5 expression in a similar manner as the induction of ISGF3 subunits in MC38 cells and human organoids (Fig. 5, *B* and *C*), it notably failed to induce *Otud5* mRNA levels (Fig. 5*D*), suggesting a nontranscriptional mechanism. Consistent with that, blocking JAKs with a pan-JAK inhibitor pyridine six did not affect the upregulation of OTUD5 by IFNγ, but did suppress the upregulation of STAT1, two and IRF9 as expected (Fig. 5*E*). It is known that OTUD5 needs to be phosphorylated in order to be active (40) and the activation of protein kinase C in T cells was shown previously to phosphorylate the deubiquitinase and to enhance its own stability (41). Thus, it is likely that IFNγ, by activating protein kinase C (41) or serine protein kinases other than JAKs, activates and stabilizes OTUD5, leading to the upregulation of its expression. We therefore treated MC38 cells with a broad protein kinase inhibitor staurosporine (STS) and examined the expression of OTUD5. As shown in Figure 5*F*, staurosporine abolished IFNγ-induced upregulation of OTUD5 and the addition of the proteasome inhibitor MG132 (2 h prior to staurosporine) restored the expression. MG132 not only restored IFNγ-induced upregulation of OTUD5, but also increased its levels before the induction, indicating that the deubiquitinase undergoes fast turnover. Taken together, these results strongly suggest that IFNγ signaling operates at two fronts, one to induce *STAT1*, *two* and *IRF9 via* the activation of JAKs and another to stabilize OTUD5 in a JAK-independent manner. The latter is necessary for the late phase of IFNγ signaling to work as OTUD5 is somehow required for ISGF3 to function (see below).

As STAT1/2 and IRF9 were induced by IFNγ, the formation of ISGF3 complex could be detected (Fig. S4*C*), and so could the *Ccl8* expression (Fig. 5*G*). The induction of *Ccl8* expression by IFNγ could also be observed in CT26 cells (another IEC line derived from colon adenocarcinoma) as well (Fig. S4*D*). To demonstrate the it is the ISGF3 that activates *Ccl8* expression, we depleted the expression of *Stat1*, *two* or *Irf9* in MC38 cells and examined *Ccl8* expression. As expected, in all three depletions, IFNγ-induced *Ccl8* expression attenuated (Figs. 5*H* and S4*E*). It is known that ISGF3 activates the expression of genes containing IFN-stimulated response element (ISRE) in their promoters (33). Examination of *Ccl8* promoter showed the presence of two potential ISREs located in the region between −250 and −1 (Fig. 5*I*). This region is responsive to IFNγ stimulation in the luciferase assay (Fig. 5*J*) and the functional ISRE was determined to be from −79 to −72 in *Ccl8* promoter (Fig. 5, *I* and *J*). Further, as seen in *Otud5-*deficient IECs, depletion of *Otud5* also attenuated the induction of *Ccl8* by IFNγ (Figs. 5*K* and S4*F*), again indicating that the IFNγ-ISGF3 signaling requires the function of *Otud5.*

To look into the functional importance of IFNγ-induced *Ccl8* expression in recruiting immune cells, we established two *Stat2* KO MC38 cell lines through CRISPR/Cas9-mediated gene editing (Fig. S4*G*) and showed that IFNγ could no longer induce *Ccl8* expression in these cells (Fig. S4*H*). Transwell chemotaxis assays demonstrated that IFNγ could enhance the migration of RAW264.7 cells (a murine monocyte cell line) toward MC38 cells (Fig. 5, *L* and *M*). While such an enhancement was abolished in *Stat2-*deficient cells, it could be largely restored by the addition of exogenous CCL8 (Fig. 5, *L* and *M*), reinforcing the role of CCL8 in immune cell recruitment as seen previously *in vivo* (Fig. 3).

### OTUD5 is a deubiquitinase of STAT1 and STAT2

**Figure 6:**
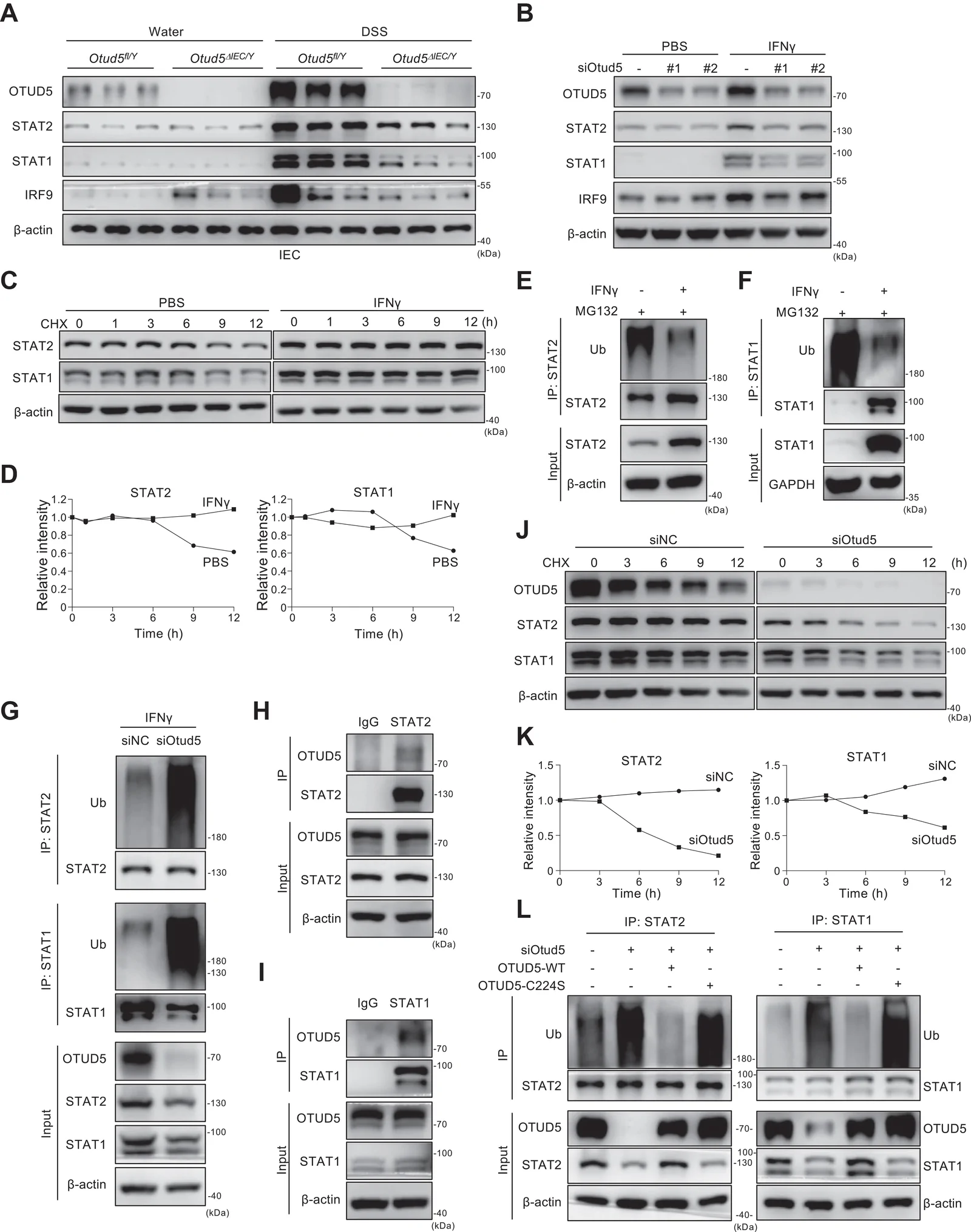
**OTUD5 regulates the stability and expression of STAT2 and STAT1.***A,* Western blot analysis in IECs from *Otud5*^*fl/Y*^ or *Otud5*^*ΔIEC/Y*^ mice treated with or without DSS. *B,* Western blot analysis in MC38 cells depleted of *Otud5* and treated with IFNγ (50 ng/ml) for 6 h. *C,* Western blot analysis of STAT2 and STAT1 expression levels in MC38 cells. The cells were treated by IFNγ (50 ng/ml) for 6 h first and then IFNγ (50 ng/ml) and cycloheximide (10 μg/ml) for the indicated times. *D*, Quantitation of STAT2 and STAT1 in *(C)*. *E and F,* ubiquitination assay of STAT2 *(E)* and STAT1 *(F*). STAT2 or STAT1 was immunoprecipitated from MC38 cells treated with IFNγ (50 ng/ml) as well as MG132 (10 μM) for 16 h. *G*, ubiquitination assay of STAT2 and STAT1. STAT2 or STAT1 was immunoprecipitated from MC38 cells transfected with siNC or siOtud5 for 48 h followed by IFNγ (50 ng/ml) treatment for 6 h *H and I,* immunoprecipitation analysis of STAT2 *(H)* and STAT1 *(I)*. STAT2 or STAT1 was immunoprecipitated from MC38 cells treated with IFNγ (50 ng/ml) for 12 h and the immunoprecipitates were analyzed with Western blotting. *J,* Western blot analysis of STAT2 and STAT1 expression levels in MC38 cells. The cells were transfected with siOtud5 for 42 h followed by IFNγ (50 ng/ml) treatment for 6 h first and then IFNγ (50 ng/ml) and cycloheximide (10 μg/ml) for the indicated times. *K,* quantitation of STAT2 and STAT1 in *(J)*. *L,* ubiquitination assay of STAT2 and STAT1. MC38 cells were overexpressed with WT OTUD5 (OTUD5-WT) or C224S mutated OTUD5 (OTUD5-C224S) and transfected with siOtud5 for 48 h followed by IFNγ (50 ng/ml) treatment for 6 h. DSS, dextran sulfate sodium; IEC, intestine epithelial cell; IFNγ, interferon gamma.

While OTUD5 is induced along with ISGF3 components by IFNγ, the deubiquitinase is actually required for ISGF3 function (Fig. 5*K*), and this requirement most likely underlies the resistance to DSS-induced colitis in *Otud5*^*ΔIEC/Y*^ mice (Fig. 3). To delineate potential mechanisms behind the requirement, we first looked at the expression of ISGF3 components in IECs isolated from *Otud5*^*fl/Y*^ and *Otud5*^*ΔIEC/Y*^ mice treated with DSS. In agreement with the immunohistochemistry (IHC) staining results (Figs. 1*D* and 4*C*), DSS treatment induced strong expression of OTUD5 and STAT2 as well as STAT1 and IRF9 in *Otud5*^*fl/Y*^ mice (Fig. 6*A*). In contrast, such induction was much attenuated in the absence of *Otud5* (Fig. 6*A*). Similarly, the induction of STAT1/2 by IFNγ was strongly suppressed in *Otud5*-depleted MC38 (Fig. 6*B*), CT26 (Fig. S5*A*) and HCT116 cells (Fig. S5*B*). Interestingly, the induction of IRF9 by IFNγ seemed independent of *Otud5 in vitro* (Figs. 6*B*, and S5, *A*, *B*). Nonetheless, the ISGF3 expression and function are dependent on *Otud5.*

Since OTUD5 is a deubiquitinase, it is unlikely required for the transcriptional upregulation of *Stat1* and *two* by IFNγ. Indeed, IFNγ could still induce mRNA expression of *Stat1, two* and *Irf9* in MC38 cells depleted of *Otud5* (Fig. S5*C*), strongly suggesting that *Otud5* works in a posttranscriptional manner. We therefore postulated that OTUD5 as a deubiquitinase maintains the stability of STAT1/2 by preventing their ubiquitination and degradation. To test that, we first examined the protein stability and ubiquitination levels of STAT1/2 following stimulation with IFNγ. Consistent with our hypothesis, we observed that IFNγ significantly enhanced the protein stability of STAT1 and 2 (Fig. 6, *C* and *D*) and suppressed their ubiquitination (Fig. 6, *E* and *F*). Consistent with its independence of *Otud5 in vitro*, the ubiquitination status of IRF9 was unaffected by IFNγ (Fig. S5*D*). Notably, the suppression of ubiquitination of STAT1/2 by IFNγ diminished in *Otud5-*depleted cells (Fig. 6*G*), suggesting that OTUD5 is a deubiquitinase for these two TFs. In support of that, an interaction between OTUD5 with STAT1 and two could be detected (Fig. 6, *H* and *I*). Further, in *Otud5-*depleted MC38 cells, STAT1 and two became very unstable (Fig. 6, *J* and *K*), and the proteasome inhibitor MG132 could restore STAT1/2 expression levels in *Otud5-*depleted cells (Fig. S5*E*). In addition, only the WT but not the catalytically inactive mutant *OTUD5* (C224S) could suppress the ubiquitination of STAT1 and two and restore their expression in *Otud5-*depleted MC38 cells (Fig. 6*L*). Taken together, these results indicate that OTUD5 maintains the stability and expression of STAT1 and two by preventing their ubiquitination and proteasomal degradation, thereby sustain IFNγ-ISGF3 signaling.

### Identification of OTUD5 inhibitor

**Figure 7:**
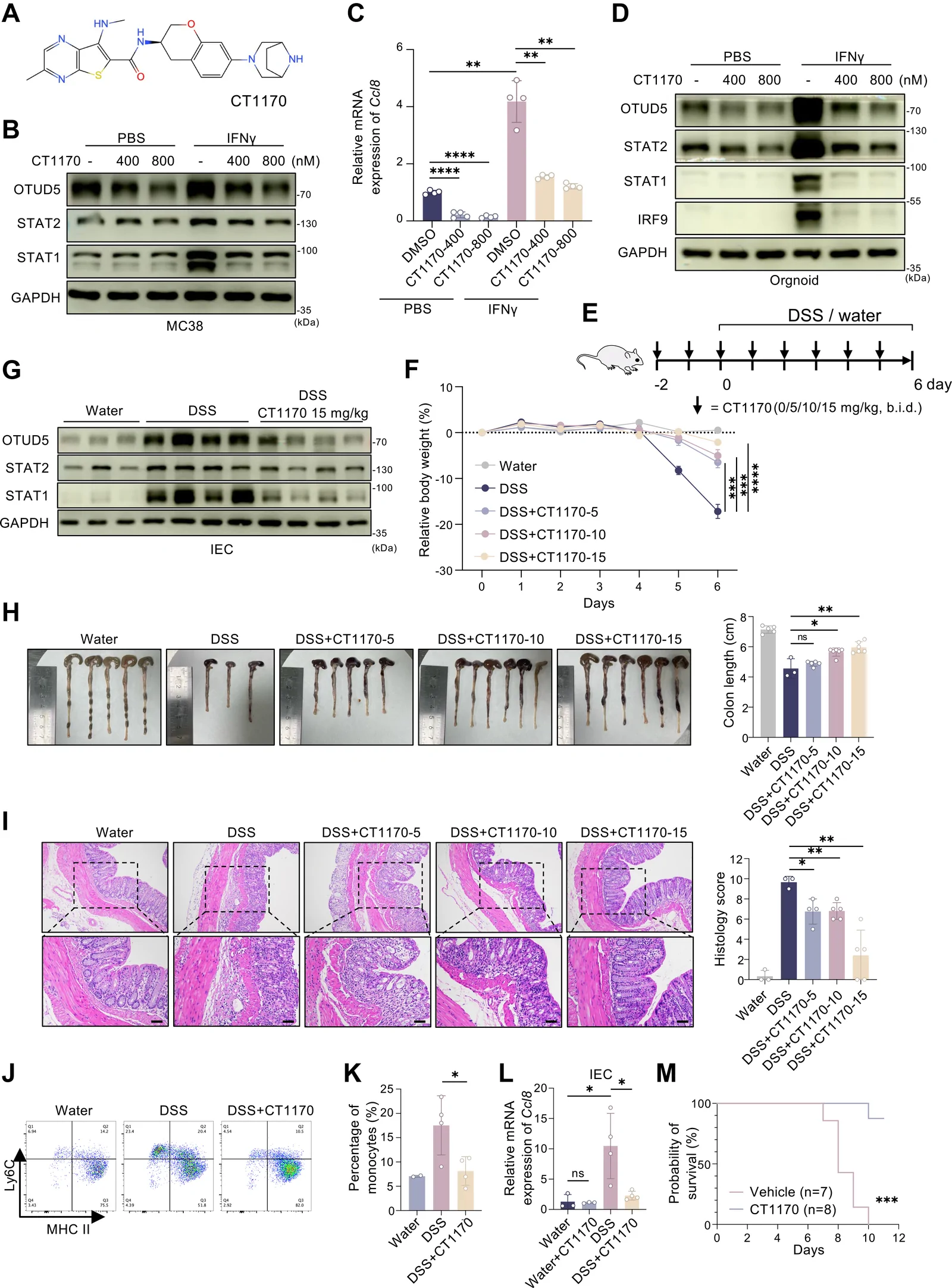
**OTUD5 inhibitor alleviates DSS-induced colitis in a dose-dependent manner.***A,* the chemical structure of CT1170. *B,* Western blot analysis in MC38 cells that were treated with or without IFNγ (50 ng/ml) plus CT1170 for 6 h *(C)* qPCR analysis of *Ccl8* mRNA expression levels in MC38 cells. The cells were treated with or without IFNγ (50 ng/ml) plus CT1170 for 24 h. *D,* Western blot analysis in human-derived intestinal epithelial organoids that were treated with or without IFNγ (50 ng/ml) plus CT1170 for 24 h. *E,* schematic diagram of CT1170 administration in DSS-induced colitis model. *F,* Western blot analysis in IECs from each treatment group (Water, DSS, and DSS plus 15 mg/kg 1170) on day 4. *G,* body weight measurements of the mice during DSS treatment *(E)* (n = 5 for Water, DSS, and DSS + CT1170-5; n = 6 for DSS + CT1170-10 and DSS + CT1170-15). 1170-5/10/15 represent 5/10/15 mg/kg of CT1170, bid. *H,* the colons were isolated from the mice in different treatment groups on day 6. The colon lengths were measured and presented. Sample sizes are identical to those in *(F)*, with n = 3 for the DSS group. *I,* H&E staining and scoring of the colons in *(H)* (n = 3 for Water and DSS; n = 4 for DSS + CT1170-5; n = 5 for DSS + CT1170-10 and DSS + CT1170–15). The scale bar represents 100 μm. *J,* flow cytometric analysis of Ly6C^+^MHC II^-^ inflammatory monocytes in the LP. Representative flow results from 4-days water-, DSS- or DSS plus CT1170 (15 mg/kg)-treated mice are shown (n = 2 for Water; n = 4 for DSS and DSS + CT1170). *K,* quantitation of the Ly6C^+^MHC II^-^ monocytes *(J)* in the LP from all animals. *L,* qPCR analysis of *Ccl8* mRNA expression levels in IECs from 4-days water-, DSS- or DSS plus CT1170 (15 mg/kg)-treated mice (n = 3 per group for water controls and n = 4 per group for DSS-treated mice). *M,* Kaplan-Meier survival analysis of the mice administered with DSS-containing water and treated with CT1170 (15 mg/kg, bid) continuously from day −2 till the end (n = 7 for DSS + vehicle; n = 4 for DSS + CT1170). Data are presented as mean ± SD (except in *G*, mean ± S.E.M. was presented). ∗, *p* < 0.05; ∗∗, *p* < 0.01; ∗∗∗, *p* < 0.001; ∗∗∗∗, *p* < 0.0001. DSS, dextran sulfate sodium; IEC, intestine epithelial cell; LP, lamina propria.

The results so far suggest that OTUD5 is a strong candidate as a target for IBD therapeutics. Inhibiting OTUD5 could potentially relieve IBD symptoms, especially in patients who are nonresponsive to anti-TNFα therapy. Identifying small molecule OTUD5 inhibitors would be the first step toward that direction. We therefore started a search for inhibitors of the DUB by screening a handful commercially available and home-made compounds (mostly USP28 inhibitors (42)) at first. Fortunately, a home-made compound CT1170 (Fig. 7*A*), structurally very close to CT1113 (42) and also a USP25/28 inhibitor as CT1113, showed strong inhibitory activity against OTUD5 in a DUB assay with an estimated IC_50_ of 147.6 nM (Fig. S6*A*), indicating a potent inhibitory capacity of CT1170 against OTUD5. Molecular modeling showed that the inhibitor binds to the activity center of OTUD5 (Fig. S6*B*). As expected, inhibiting OTUD5 with CT1170 enhanced the ubiquitination modification on STAT1 and 2 (Fig. S6*C*).

Having identified a OTUD5 inhibitor, we first wanted to test its effectiveness in suppressing IFNγ signaling. In MC38 cells, CT1170 treatment blocked IFNγ-induced up-regulation of STAT1 and 2 (Fig. 7*B*) and the expression of *Ccl8* (Fig. 7*C*). In the colon organoids derived from one IBD patient, the compound effectively blocked IFNγ signaling as evidenced by the diminished upregulation of all three ISGF3 components (Fig. 7*D*). Similar suppression of IFNγ-induced upregulation of STAT1 and two was observed in CT26 cells (Fig. S6*D*) and HCT116 cells (Fig. S6*E*). CT1170 also blocked induction of *Ccl8* expression by IFNγ in CT26 cells too (Fig. S6*F*). Of note, the compound also induced downregulation of OTUD5 in murine and human intestinal cell lines as well as in human colon organoids, likely because OTUD5 acts as its own deubiquitinase. Given that CT1170 can also inhibit both USP25 and USP28, we examined the effect of depleting the expression of these two DUBs. As shown in Fig. S6*G*, depleting either one of them or both of them simultaneously had little effect on STAT1 and two levels, indicating that the effect on STAT1 and two by CT1170 most likely goes through OTUD5.

### Inhibiting OTUD5 alleviates DSS-induced colitis

Next, we wanted to test the efficacy of CT1170 in relieving DSS-induced colitis. Male adult mice were administered with CT1170 by oral gavage, twice daily, at dosages of 5, 10, and 15 mg/kg, starting 2 days prior to the administration of DSS-containing drinking water. CT1170 treatment was continued until the sixth day of DSS administration when the mice were sacrificed for analysis (Fig. 7*E*). As expected, the compound suppressed the induction of OTUD5, STAT1 and two in the IECs by DSS treatment (Fig. 7*F*). Over the course of DSS administration, it became obvious that the mice treated with CT1170 lost much less body weight than the control animals, and even more strikingly, those given 15 mg/kg lost little or no body weight at all (Fig. 7*G*). When the colon was dissected out and compared at the end of DSS administration, the mice treated with CT1170 showed dose-dependent improvements (Fig. 7*H*). Histological examination of the colon showed that CT1170 prevented tissue destruction induced by DSS in a dose-dependent manner (Fig. 7*I*). Further, the recruitment of Ly6C^+^MHCII^-^ monocytes diminished in CT1170-treated mice (Fig. 7, *J* and *K*), likely because the expression of *Ccl8* in the IECs was suppressed by the compound (Fig. 7*L*). Consistent with the alleviation of DSS-induced colitis, CT1170 could prolong the survival of mice under continued DSS treatment (Fig. 7*M*).

To further verify that the small-molecule inhibitor CT1170 exerts its effects by suppressing the IFNγ signaling pathway, we performed functional rescue experiments in the DSS colitis model. We treated the mice with DSS and CT1170, and gave CCL8 to these mice on day 4 *via* intravenous injection and five *via* intraperitoneal injection (Fig. S7*A*). The mice were sacrificed on day 7 for analyses. It was obvious that exogenous CCL8 significantly reduced the efficacy of CT1170 as indicated by the increased weight loss (Fig. S7*B*), shortened colon length (Fig. S7*C*), and worsened histological scores (Fig. S7*D*) in CCL8-administered mice than in nonCCL8 mice. More importantly, supplementing CCL8 restored the infiltration of Ly6C^+^MHCII^-^ monocytes in CT1170-treated mice (Fig. S7*E*). These results are in agreement with the rescuing experiment performed in *Otud5* CKO mice (Fig. 3), reinforcing the notion that *Ccl8* induced by IFNγ-ISGF3 signaling promotes intestinal inflammation in the DSS-induced murine IBD model.

### OTUD5 inhibitor suppresses colitis-associated tumorigenesis

**Figure 8:**
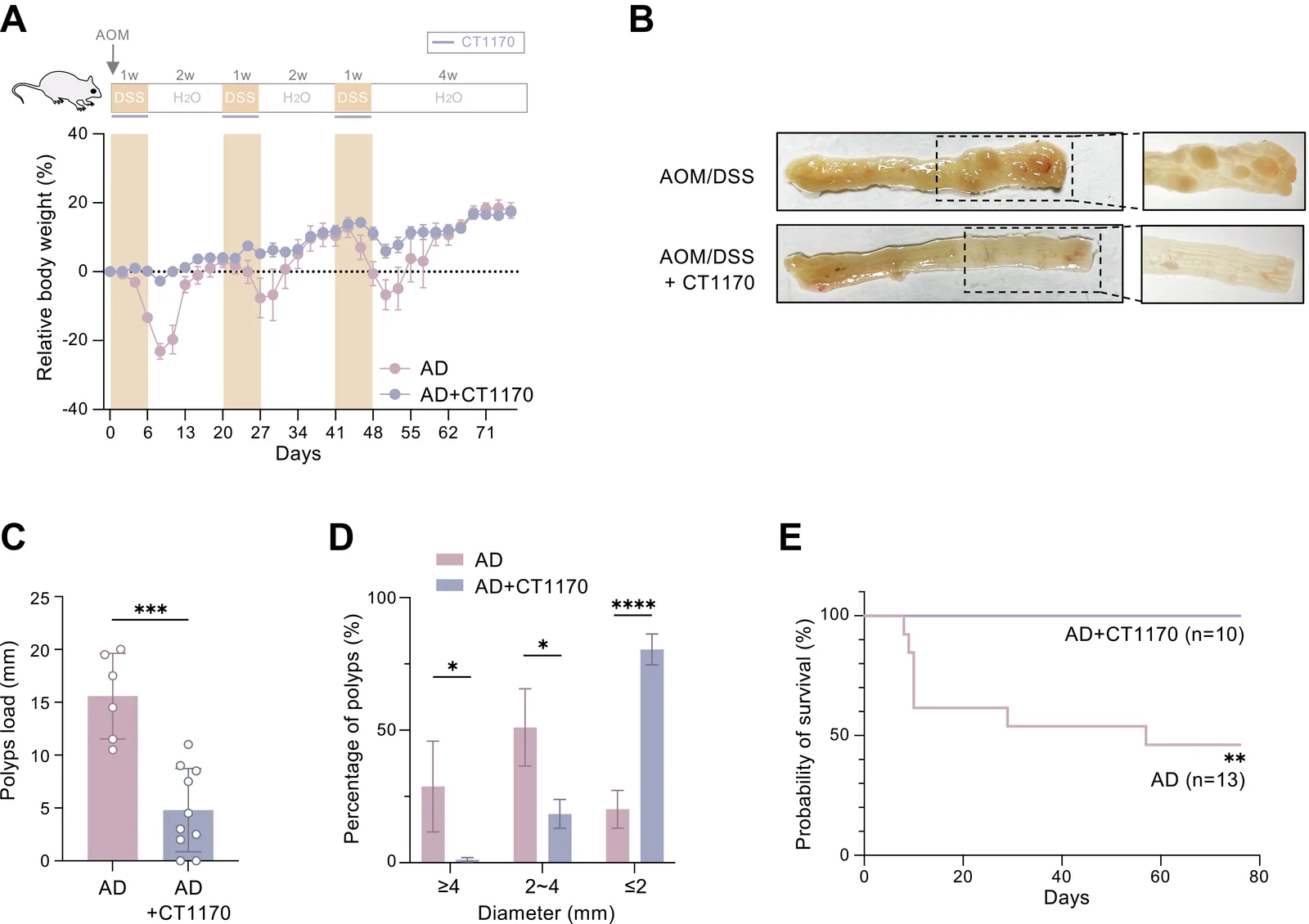
**Pharmacologic inhibition of OTUD5 suppresses colitis-associated tumorigenesis.***A,* schematic diagram of the experiment *(top)* and body weight measurements of the mice during the experiment *(bottom)* (n = 13 for AD; n = 10 for AD + CT1170). *B,* representative images of polyps in distal colon from each treatment group. *C,* bar graph of the polyps load in each group (AD: n = 6 remaining due to mortality; AD + CT1170: n = 10). *D,* bar graph of the polyps divided by size. *E,* Kaplan-Meier survival analysis of the mice from *(A)*. Data are presented as mean ± SD (except in *A,* mean ± S.E.M. was presented). ∗, *p* < 0.05; ∗∗, *p* < 0.01; ∗∗∗, *p* < 0.001; ∗∗∗∗, *p* < 0.0001.

Patients with long-standing IBD are at a higher risk of developing colorectal cancer, highlighting the contributing role played by inflammation in tumorigenesis (43). The inflammation-accelerated tumorigenesis can be recapitulated by repeated inflammation cycles through DSS administration in combination with one-time administration of AOM at the beginning (AOM/DSS model of colitis-associated cancer) (44). To explore the potential of CT1170 in preventing tumorigenesis in this model, we subject mice to the AOM/DSS scheme with or without CT1170 (at 15 mg/kg, b.i.d.) covering all DSS cycles (Fig. 8*A*). Consistent with the observation above, CT1170 prevented the loss of body weight associated with each DSS cycle (Fig. 8*A*). At the end of the AOM/DSS scheme, we examined the burden of tumors in the intestine (Fig. 8*B*). It was obvious that the mice received CT1170 treatment displayed much fewer (Fig. 8*C*) and much smaller (Fig. 8*D*) intestinal polyps. Moreover, over the course of the AOM/DSS scheme, more than 50% of the nonCT1170 group of mice had died, while none had died in the CT1170 group (Fig. 8*E*).

## Discussion

A significant subset of IBD patients fail to respond to anti-TNFα therapy, yet the molecular mechanisms driving this primary nonresponse remain poorly understood. While current literature has extensively explored immune cell-intrinsic pathways, the role of IECs and post-translational regulatory nodes—specifically ubiquitination—in sustaining therapy-resistant inflammation is less explored. Through a combination of patient biopsies and IEC-specific KO mice, our findings indicate that the deubiquitinase OTUD5 serves as an important contributor to therapy-resistant intestinal inflammation.

Prior work by Dinallo *et al.* noted an increase of OTUD5 in the diseased LP of IBD patients (20). Notably, their immunohistochemical data also showed that OTUD5-expressing cells were diffusely present within the epithelial compartment, though their functional study focused on the LP (20). To ensure the validity of our observation, we confirmed antibody specificity using both negative controls and tissue sections from IEC-specific *Otud5* knockout mice, ruling out nonspecific epithelial staining (Fig. S1, *A* and *B*). We also observed that while high concentrations of the primary antibody yield positive signals in the LP, the staining intensity is consistently stronger in the epithelial layer. This indicates a higher expression of OTUD5 in IECs. Consequently, at lower optimized working concentrations, the abundant epithelial OTUD5 is preferentially stained, explaining the pattern seen in our figures. Of note, the rabbit polyclonal anti-OTUD5 antibody used in our study (Biorbyt) differs from the polyclonal antibody used by Dinallo *et al.* (Thermo Fisher Scientific). Because the specific catalog number and immunogen details were not reported in their study, we cannot rule out the possibility that differences in targeted epitopes, alongside optimized working concentrations, contributed to the variations in compartment-specific staining intensities between the two works. By using IEC-specific *Otud5* KO mice, our study defines the cell-intrinsic necessity of epithelial OTUD5 in chronic colitis, complementing the known roles of this deubiquitinase in the LP.

OTUD5 expression is elevated in patients with IBD, especially in those who do not respond to anti-TNFα therapy (Fig. 1). We demonstrate that the upregulation of OTUD5 is necessary for STAT1 and two protein stability and expression, therefore sustaining IFNγ-ISGF3 signaling. ISGF3 in turn drives the expression of cytokines/chemokines in IECs which recruit immune cells to cause inflammation and colitis. We showed that CCL8 (CCL18 in human) is a target of ISGF3 and necessary for the recruitment of Ly6C^+^ inflammatory monocytes to the intestine in mice.

Given that both TNFα and IFNγ are produced along with many other cytokines/chemokines in the colon in IBD patients (9) and the anti-TNFα therapy NRs seemed to express higher levels of IFNγ (Fig. S4*A*), it is thus reasonable to believe that IFNγ-high IBD patients are less responsive to anti-TNFα antibodies as their diseases is driven more by IFNγ-ISGF3 signaling than by TNFα signaling. Indeed, increased expression of IFN signature genes has been found to be associated with anti-TNFα treatment nonresponsiveness (45, 46). It is worth noting that all three components of ISGF3, STAT1, STAT2, and IRF9, are required for the induction of colitis by DSS treatment in mice (30, 32, 47), highlighting the importance of the genes activated by ISGF3 in the development of IBD. As JAKs are required to activate STATs including STAT1 and STAT2 (and thus the formation of ISGF3), our findings here lend further support for the clinical application of JAK inhibitors in IBD. However, because JAKs are required to activate all STATs, it is not surprising that their inhibitors have broad side effects, limiting their clinical applicability in IBD (48, 49, 50). Thus, other targeting strategies with less broad effects as inhibiting JAKs are worth exploring.

IFNγ induces robust expression of OTUD5 to stabilize STAT1 and 2 (Figs. 5 and 6). Interestingly, the induction of the deubiquitinase is not at the transcriptional level (Fig. 5*D*) and is JAK-independent (Fig. 5*E*). Rather, OTUD5 undergoes IFNγ-mediated and protein kinase-dependent self-stabilization. Thus, IFNγ activates ISGF3 in two ways. One is transcriptional and JAK-dependent that stimulates the mRNA expression of *STAT1, two* and *IRF9*. Another is post-transcriptional and JAK-independent that stabilizes the deubiquitinase OTUD5 to prevent the nascent STAT1 and two from being ubiquitinated and degraded. Interestingly, unlike in the tumor-derive intestinal cell lines, the induction of IRF9 in the IECs of DSS-treated mice and in IFNγ-treated human colon organoids also depends on OTUD5, suggesting IRF9 might also need OTUD5 to sustain its protein stability in primary cells. Nonetheless, the post-transcriptional arm of IFNγ signaling is critical for ISGF3 to form and function, offering a unique opportunity to limit the late phase of IFNγ responses *via* blocking OTUD5. Indeed, compromising the function of OTUD5 through knocking down in cells or KO in mice could substantially weaken IFNγ-ISGF3 signaling, as evidenced by the reduction in the expression of *Ccl8* and amelioration of DSS-induced colitis (Figs. 2 and 5). The fact that inactivation of the deubiquitinase in IECs alone could relieve colitis indicates that IECs themselves are important producers of inflammatory signals. Our work thus uncovered a regulatory loop that sustains IFNγ-ISGF3 signaling and identifies OTUD5 as a potential target for IBD treatment, especially for the treatment of anti-TNFα therapy NRs.

The deubiquitinase OTUD5 has been considered as a negative regulator of innate immunity. While early work showed OTUD5 limits type I interferon by deubiquitinating TNF receptor-associated factor 3 (51), recent evidence—including our own—indicates that OTUD5 functions as a critical pro-inflammatory scaffold and stabilizer across diverse signaling cascades. In the context of sepsis and systemic toll-like receptor activation, OTUD5 was shown to promote MyD88 oligomerization (22), thereby fueling the "cytokine storm" characteristic of septic shock. This pro-inflammatory mechanism is strikingly mirrored in the intestinal epithelium (IECs) during chronic colitis. We demonstrate that in the intestinal epithelium, OTUD5 is upregulated and stabilizes STAT1 and 2, which in turn sustains the IFNγ-ISGF3 axis. This stabilization is critical for the transcriptional upregulation of *Ccl8* and the subsequent recruitment of pathogenic Ly6C^+^ inflammatory monocytes (Fig. 3).

By demonstrating the role of OTUD5 in ISGF3-mediated intestinal inflammation, alongside its established function in MyD88-mediated systemic inflammation, our study highlights this deubiquitinase as an important shared regulatory node in hyper-inflammatory processes. Specifically, while the OTUD5-STAT1/2 axis serves as a major driver of IFNγ-mediated inflammation, it remains highly plausible that other potential substrates of OTUD5 co-contribute to barrier dysfunction or downstream immune signaling, a possibility that warrants future unbiased proteomic screening.

In this study, we identified a small-molecule compound, CT1170, which exhibits potent inhibitory activity against OTUD5 (Fig. 7). While CT1170 administration significantly alleviates DSS-induced inflammation and colitis-associated tumorigenesis, these results must be interpreted with caution given the preliminary pharmacological characterization of the compound and the potential contribution of unmapped off-target effects on other cellular pathways or related DUBs. CT1170 is closely related to CT1113, a potent USP25/28 inhibitor (42), and our unpublished data indicate that CT1170 is also a potent USP25/28 inhibitor. Given that CT1170 inhibits multiple DUBs, its protective efficacy in DSS-induced colitis may involve the concurrent suppression of USP25 and USP28. However, our genetic knockdown of USP25 and USP28 (Fig. S6*G*) indicates that the disruption of the STAT1/2-ISGF3 cascade is driven primarily by OTUD5 inhibition. The broad systemic protection observed with CT1170 likely reflects its distribution across both the IECs and LP immune cells. Developing epithelial-targeted formulations will be necessary to isolate epithelial-specific effects and optimize targeting efficiency.

The identification of the small-molecule compound CT1170 offers a promising pharmacological starting point to modulate these over-activated signaling cascades. Mechanistically, blocking downstream ISGF3 assembly *via* OTUD5 while leaving upstream JAK kinetics intact provides a targeted approach that avoids the generalized signaling blockades typical of broad-spectrum JAK inhibitors. Although further optimization is strictly required to refine its specificity over related DUBs and to establish localized delivery, targeting the OTUD5-associated pathway represents a potential therapeutic strategy for patients refractory to current standard-of-care biologics.

## Experimental procedures

### Human tissue samples

This study retrospectively included 20 normal colon tissue samples (from the excision of intestinal polyps) and 37 tissue samples from IBD patients divided to three groups according to their responsiveness to anti-TNFα therapy: responders (n = 12), primary NRs (n = 6), secondary NRs (n = 9), and unknown (n = 10). Paraffin-embedded colon sections from patients with IBD were obtained from the First Affiliated Hospital, Zhejiang University School of Medicine. The use of human colon tissue samples in this study was approved by the Ethics Committee of the First Affiliated Hospital, Zhejiang University School of Medicine (No. 2023-179), and conducted according to the guidelines of the Declaration of Helsinki.

### Human colonic organoids

Human colonic organoids were established and maintained using a serum-free Human Colonic Organoid Kit (K2003-HC, bioGenous) according to the manufacturer's protocol. Briefly, colonic tissue biopsies obtained with informed consent were minced and digested with Tissue Digestion Solution at 37 °C for 5 to 30 min. The resulting cell clusters were filtered through a 100 μm strainer, washed, and embedded in growth factor-reduced Organoid Culture ECM (M315066, bioGenous). The suspension was seeded as 30 μl droplets in 24-well plates and allowed to polymerize at 37 °C for 15 to 25 min. Organoids were initially cultured in Primary Culture Medium and subsequently transitioned to Maintenance Medium for long-term expansion. Cultures were maintained in a humidified incubator at 37 °C with 5% CO_2_ with medium replenishment every 3 days. For passaging, organoids were harvested every 5 to 8 days and dissociated into small clusters using Organoid Dissociation Solution (E238001) or mechanical disruption before reseeding in fresh ECM.

### Analysis of human patient datasets

The datasets of IBD patients who were NR or responder to IFX were obtained from the Gene Expression Omnibus databases: GSE73661 and GSE169879 (https://www.ncbi.nlm.nih.gov/geo/). To include as much as possible of the up-regulated genes in NR group (*versus* responder group), we set *p* < 0.05 and log_2_FC > 0 as threshold using Limma v3.62.2 R package. The intersection of differential up-regulated genes from two datasets was acquired for further TF enrichment, GO and KEGG analysis. TF enrichment analysis was conducted by ChEA3 (https://maayanlab.cloud/chea3/) (29), using ENCODE ChIP-seq data. GO and KEGG analysis were performed on DAVID (https://david.ncifcrf.gov/) (52, 53).

### Animals

WT C57BL/6 mice were purchased from Hangzhou Ziyuan Laboratory Animal Technology Co., Ltd and randomly assigned to experimental groups. *Otud5*^fl/Y^ mice (C57BL/6 background) were generated by Dr Lingqiang Zhang from the State Key Laboratory of Proteomics, Beijing Proteome Research Center, Beijing Institute of Radiation Medicine. *Villin-Cre* mice (Catalog C001243, C57BL/6 background) were purchased from Cyagen/Viewsolid Biotech Co. Ltd. IEC-specific *Otud5* KO male mice (*Otud5*^*fl/Y*^, *Vil1-Cre*^*+*^) and littermate control male mice (*Otud5*^*fl/Y*^) were acquired by crossing *Otud5*^*fl/fl*^ female mice and *Villin-Cre* heterozygous male mice. All animals were housed in specific pathogen-free facilities in the First Affiliated Hospital of Zhejiang University under temperature (22 ± 2 °C) and humidity-controlled (55 ± 5%) conditions with 12-h light/12-h dark circadian cycle. All animal experiments were approved by the Animal Care and Use Committee of the First Affiliated Hospital, Zhejiang University School of Medicine (No. 2023-910).

### Acute colitis model and treatment

DSS-induced colitis model was established as described previously (54). Briefly, 6- to 10-week-old male mice were administered with 3% (w/v) DSS (MW 36,000–50,000 Da, MP Biomedicals) in the drinking water for 6∼7 days. Fresh DSS water was replaced every other day.

For CT1170 administration, the mice received oral gavage of CT1170 at the indicated concentration twice daily, starting 2 days prior to DSS administration and continuing until sacrifice.

### Colitis-associated cancer model and treatment

DSS-induced colitis-associated cancer model was established as described previously (54). Briefly, 6- to 10-week-old male mice were intraperitoneally injected with the genotoxic agent AOM, 10 mg/kg at the first day of modeling. Then the mice were administered with three cycles of treatment including 2% (w/v) DSS in the drinking water for 1 week followed by normal drinking water for 2 weeks (4 weeks in the last cycle). Polyps load was quantified as the sum of diameters of all polyps in the intestinal tract of an animal.

For CT1170 administration, the mice received oral gavage of CT1170 (15 mg/kg) twice daily during the DSS administration.

### Histopathology scores

The distal portions of mice colons were fixed with 4% paraformaldehyde. Paraffin-embedded sections (5 μm thick) were used for H&E staining. Histopathology scores were evaluated according to inflammation severity (0∼3), inflammation extent (0∼3) and crypt damage (0∼4) (55). The total score ranged from 0 (no colitis) to 10 (severe colitis).

### Isolation of IECs and LP cells

Colon tissues were excised, longitudinally cut and thoroughly washed. After being cut into 1 cm pieces, tissues were shaken twice in pre-warmed isolation buffer (HBSS containing 5% FBS, 7 mM EDTA, 1 mM DTT, 20 mM HEPES and 0.2% trypsin) at 37 °C for 15 min. Cells in the supernatant were filtered and loaded on a 20/40% discontinuous Percoll gradient and centrifuged at 800*g* for 20 min, and then IECs were collected from the interphase. The rest of the tissue pieces were digested in pre-warmed digestion buffer (HBSS containing 1 mg/ml type IV collagenase, 100 μg/ml DNase I, 10% FBS and 10 mM HEPES) with shaking at 37 °C for 45 min. Cells in supernatant were then filtered and collected as LP cells for further analysis.

### Cell culture and treatment

Cell lines MC38 (RRID: CVCL_B288), CT26 (RRID: CRL_2638) and HEK-293T (RRID: CVCL_0045) were purchased from ATCC. They were cultured in DMEM medium supplemented with 10% FBS (NFBS-1500A, Noverse) and 1× penicillin-streptomycin (BL505A, Biosharp) at 37 °C in 5% CO_2_. DMEM supplemented with 2% FBS was used for cell seeding, and DMEM supplemented with 0.5% FBS was used for IFNγ (PeproTech) stimulation. For siRNA transfection, CALNP™ RNAi *in vitro* (D-Nano Therapeutics) was used according to the manufacturer's instruction. The primer sequences are listed in Table S3. The cell lines were kept contamination free.

### RNA extraction and quantitative real-time polymerase chain reaction

Total RNA from colon tissues or cultured cells was extracted using the TRIzol (Invitrogen) following the manufacturer’s instructions, then was reversely transcribed to cDNA using Evo M-MLV RT Master Kit (AG11706, A&G). quantitative real-time polymerase chain reaction was performed using SYBR Green Pro Taq HS Kit (AG11718, A&G). The relative gene expression levels were analyzed using the 2^−ΔΔCT^ method and normalized to the expression levels of TBP. The primer sequences are listed in Table S4.

### RNA-seq

The RNA sequencing was performed by the Sequencing platform of the Central Laboratory, the First Affiliated Hospital, Zhejiang University School of Medicine. To include as much as possible of the down-regulated genes in *Otud5*^*ΔIEC/Y*^ mice, we set log_2_FC < 0 and *p* < 0.05 as the threshold.

### Immunohistochemistry

IHC was performed on 5 μm thick sections. Following conventional dewaxing in xylene, rehydration through graded alcohols, and blocking of endogenous peroxidase in 3% hydrogen peroxide, sections were boiled in citrate buffer pH 6.0 (ZLI-9064, ZSGB-BIO) for antigen retrieval. After blocking with goat serum (SL038, Solarbio), sections were incubated with rabbit anti-OTUD5 antibodies (orb185715, diluted 1:200, Biorbyt) or rabbit anti-CD45 antibodies (A2115, diluted 1:200, Abclonal) overnight at 4 °C, then further incubated with 2-step IHC Kit (PV-6001; ZSGB-BIO) at room temperature for 50 min. For negative controls, the primary antibody was omitted and replaced with antibody dilution buffer, followed by incubation with the identical secondary antibody and substrate amplification steps (Fig. S1). Sections were visualized by DAB (ZLI-9018, ZSGB-BIO) and hematoxylin. Captured images were quantified with the ImageJ (https://imagej.net/) software (v2.1.0).

### Western blotting analysis

For western blotting analysis, cells were lysed in 2× SDS buffer (BL502, Biosharp) and then heated at 100 °C for 10 min. The samples were separated on an SDS-polyacrylamide gel and transferred onto nitrocellulose membranes. The membranes were incubated in blocking buffer (5% nonfat dry milk dissolved in TBST) for 1 h, followed by overnight incubation with primary antibodies at 4 °C. After three washes with TBST, the membranes were incubated with horseradish peroxidase-conjugated secondary antibodies at room temperature for 1.5 h. Subsequently, the membranes were washed three times and visualized using SuperPico ECL Chemiluminescence Kit (E422, Vazyme). The expression of GAPDH or β-actin was used as a loading control.

### Immunoprecipitation and ubiquitination assays

For Immunoprecipitation analysis, cells were lysed in NaCl, EDTA, Tris-HCl, and NP-40 (NETN) buffer (100 mM NaCl, 1 mM EDTA, 20 mM Tris-HCl PH8.0 and 0.5%NP-40) supplemented with protease inhibitors (Roche Diagnostics) and phosphatase inhibitors. After removing debris by centrifuge, the supernatant was incubated with appropriate antibodies overnight at 4 °C, and then incubated with protein A/G-conjugated magnetic beads (PB101, Vazyme) for 30 min at room temperature. In some experiments, anti-FLAG G1 Affinity Resin (L00432, GenScript) were also used to precipitate target protein. After washing with NETN buffer for at least 3 times, beads- or resins-bound proteins were eluted by boiling in 2× SDS buffer (BL502, Biosharp).

For protein ubiquitination analysis, cells were lysed and boiled in NETN buffer supplemented with 1% SDS and 1% sodium deoxycholate. After removing debris by centrifuge, the supernatant was diluted into 0.1% SDS with NETN buffer, and precipitated and eluted as described above.

### Dual-luciferase assay

Luciferase reporter plasmids were constructed by inserting target promoter fragments into pGL4.1 plasmid. Cells were transfected with Luciferase reporter plasmids and Renilla plasmids. Dual-luciferase assay was performed using the Dual-Luciferase Reporter Assay System (Promega) following the manufacturer’s instructions. Renilla luciferase activity was used as transfection control.

### Flow cytometry

For flow cytometry analysis, cells were washed and resuspended in Flow Cytometry Staining Buffer (00-4222-26, Invitrogen). After being blocked with anti-FcR (553142, BD), cells were stained with Zombie NIR Fixable Viability Kit (423105, Biolegend) and antibodies targeting surface markers (CD45-BV785, CD11b-PE/CF594, Ly6G-FITC, Singlec-F-Super Bright 645, Ly6C-BV605 and MHC II-BV421). Data were acquired on BD LSRFortessa and analyzed with FlowJo. Gating strategy was shown in Fig. S2.

### *In vitro* deubiquitination assay

*In vitro* deubiquitination assay was performed as described previously (56). Briefly, OTUD5-FLAG was expressed and immunoprecipitated from HEK-293T cells, and run on a SDS-PAGE gel for quantitation *via* Commasie-staining. The assay was performed in the ubiquitination assay buffer (50 mM Tris-HCl pH 7.5, 1 mM EDTA, 100 mM NaCl, 0.05% CHAPS, 5 mM DTT). The inhibitor was first incubated with OTUD5 (15 nΜ) for 30 min. After adding ubiquitin-rhodamine 110 (25 nΜ, U-555, BostonBiochem), serial readings of fluorescence intensity were obtained on a MD SpectraMax i3x. The activity of OTUD5 was calculated based on the fluorescence intensity of rodamine 110 released.

### Statistics

Statistics were performed using GraphPad Prism 8 (https://www.graphpad.com/). Student's *t* test was used when comparing two groups with normal distribution and equal variance (with Welsh correction when unequal variance). Mann Whitney U test was used when comparing two groups with unequal variance. Survival curve was analyzed using a log-rank (Mantel–Cox) test. Data were displayed as mean ± SD. Differences were considered statistically significant at ∗*p* < 0.05, ∗∗*p* < 0.01, ∗∗∗*p* < 0.001, ∗∗∗∗*p* < 0.0001.

### Patient consent for publication

Not applicable.

## Ethics approval

The use of human colon tissue samples in this study was approved by the Ethics Committee of the First Affiliated Hospital, Zhejiang University School of Medicine (No. 2023-179). Participants gave informed consent to participate in the study before taking part. All animal experiments were approved by the Animal Care and Use Committee of the First Affiliated Hospital, Zhejiang University School of Medicine (No. 2023-910).

## Data availability statement

Data are available upon reasonable request.

## Supporting information

This article contains supporting information.

## Conflict of interest

The authors declare that they have no conflicts of interest with the contents of this article.
