# Supplementary material for: OTUD5 maintains STAT1/2 stability and promotes IFNγ-driven intestinal inflammation
Source: J Biol Chem. 2026 Aug 13;302(10):113420. doi: 10.1016/j.jbc.2026.113420 (PMC13559827; doi:10.1016/j.jbc.2026.113420)
Supplement: Supplementary file 1 [file mmc1.pdf]

Figure S1

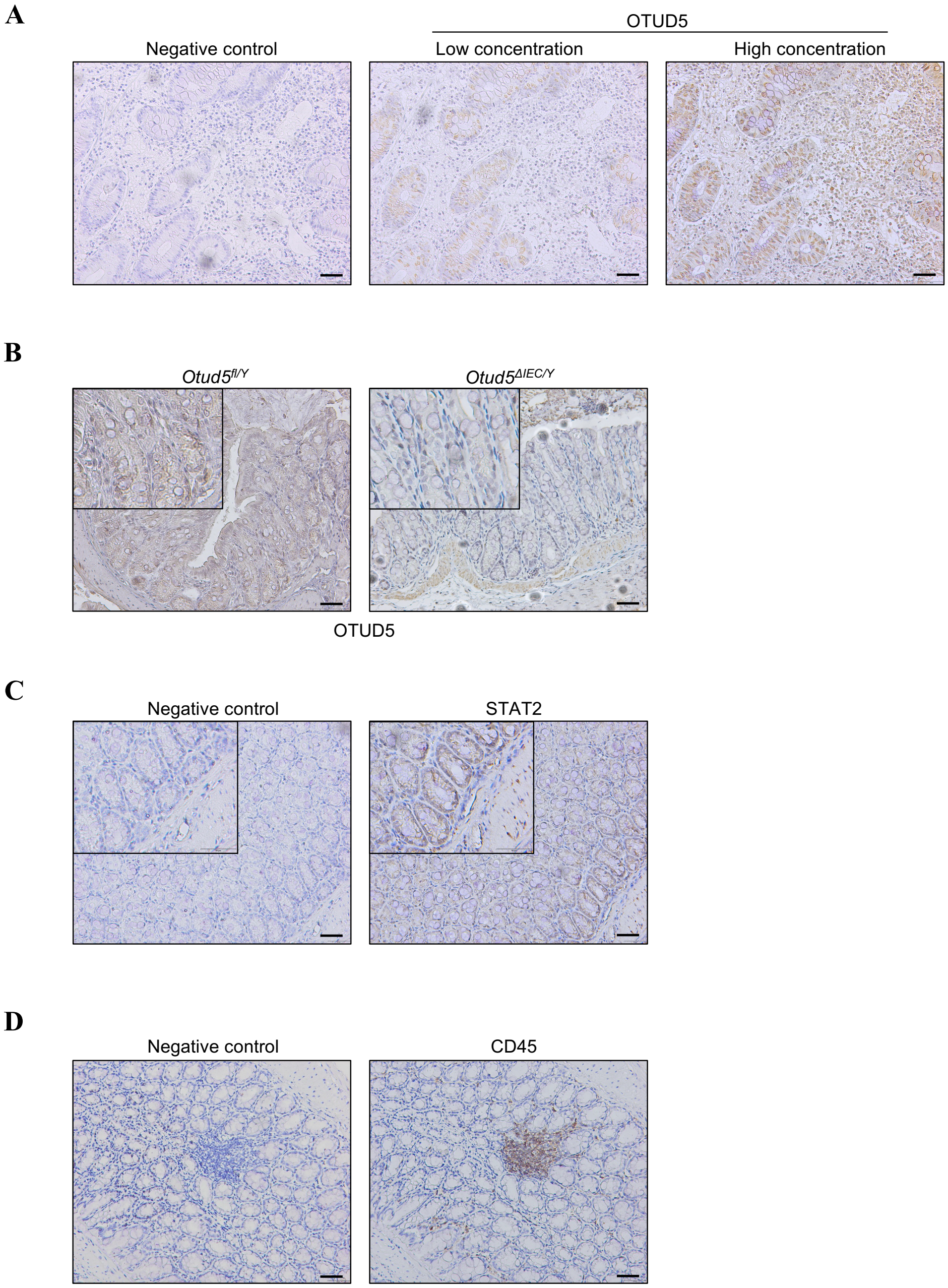

Figure S1. Validation of antibody specificity

(A) Immunohistochemistry analysis of OTUD5 in patient-derived colon tissue. Scale bar: 50 μm.

(B) Immunohistochemistry analysis of OTUD5 in colon tissue from *Otud5*$^{fl/Y}$ (3-day DSS treatment) and *Otud5*$^{\Delta IEC/Y}$ (6-day DSS treatment) mice. Scale bar: 50 μm.

(C) Immunohistochemistry analysis of STAT2 in colon tissue from C57 mice (4-day DSS treatment). Scale bar: 50 μm.

(D) Immunohistochemistry analysis of CD45 in colon tissue from C57 mice (without DSS treatment). Scale bar: 50 μm.

Figure S2

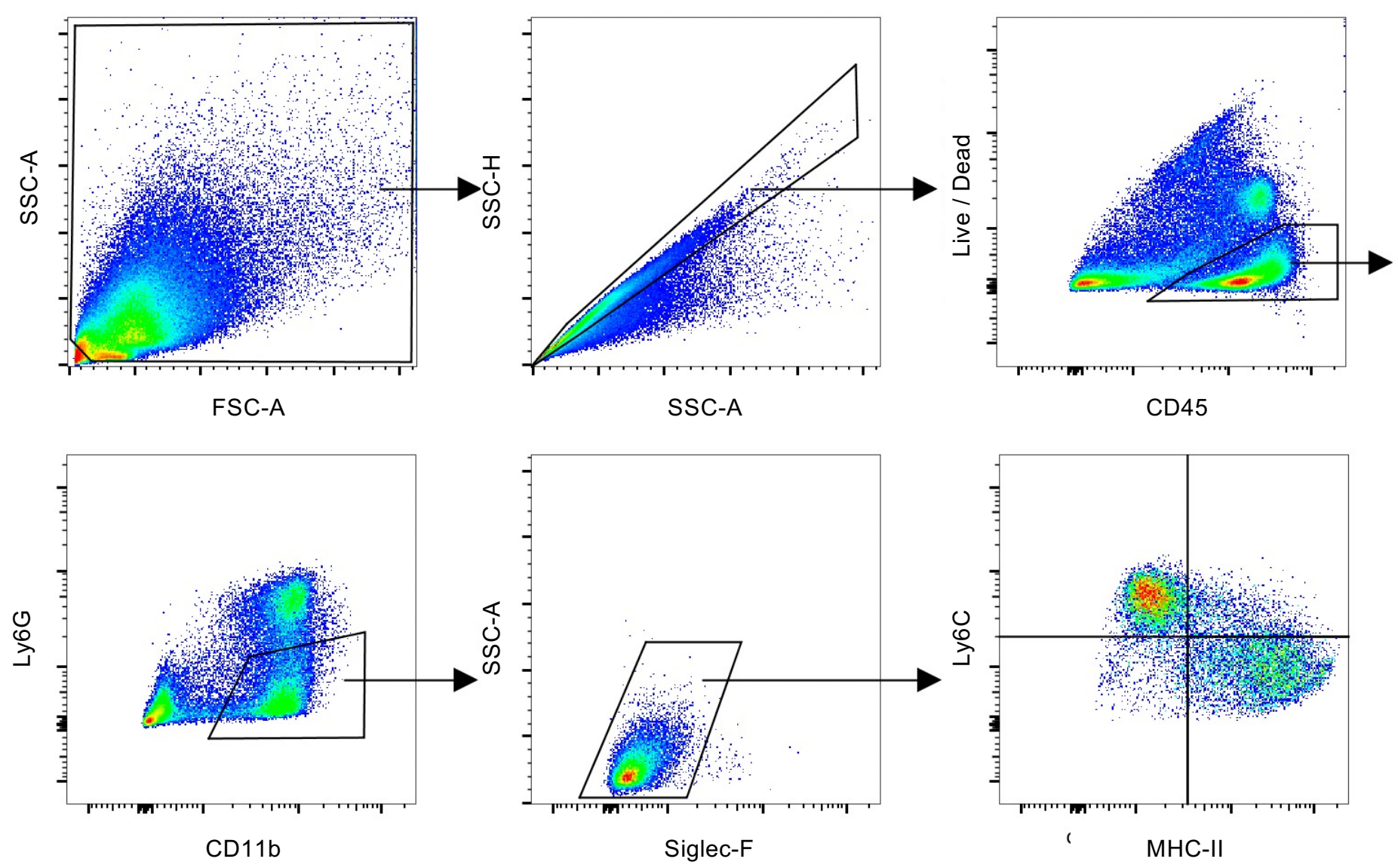

Figure S2. Gating strategy of flow cytometric analysis

Figure S3

A

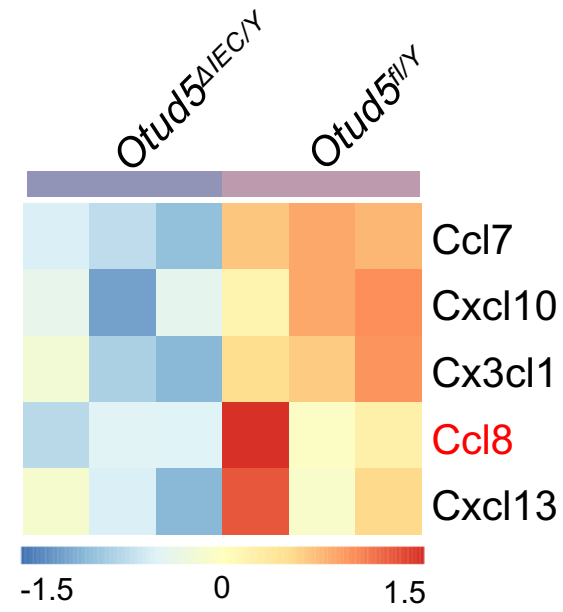

B

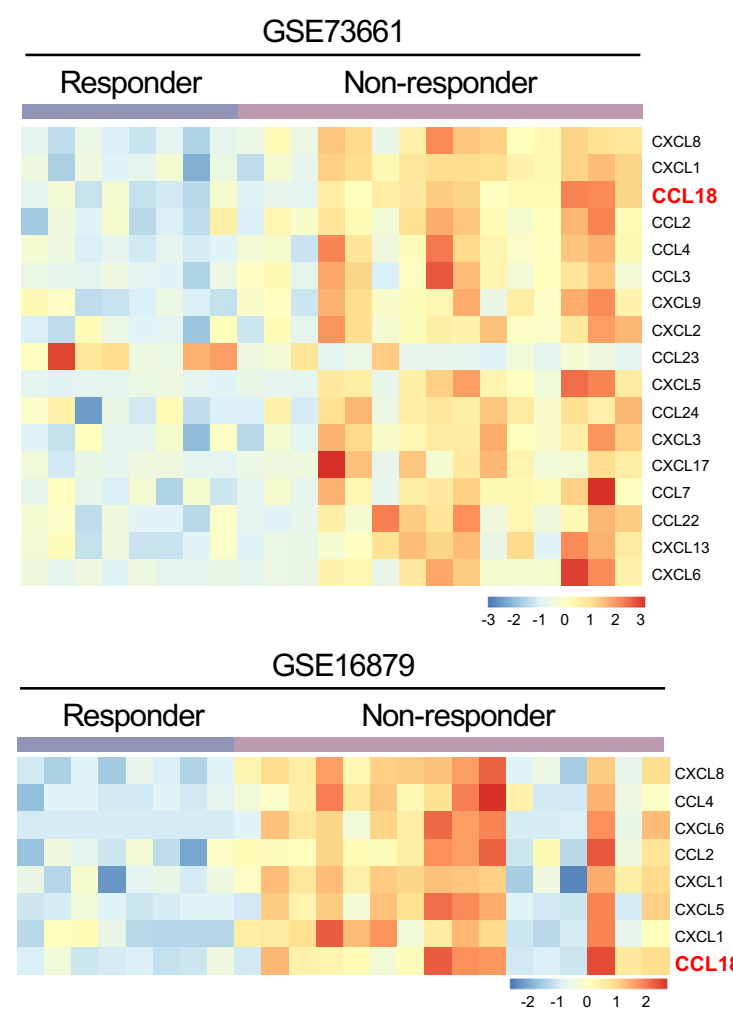

Figure S3. Heatmap of differentially expressed chemokines

(A) Differential chemokine expression profile in Otud5-deficient IECs. Heatmap displaying RNA-seq results of chemokines from *Otud5*$^{fl/Y}$ and *Otud5*$^{\Delta IEC/Y}$ mice after 4 days of DSS administration.

(B) Heatmap of differentially expressed chemokines from GSE73661 and GSE16879.

Figure S4

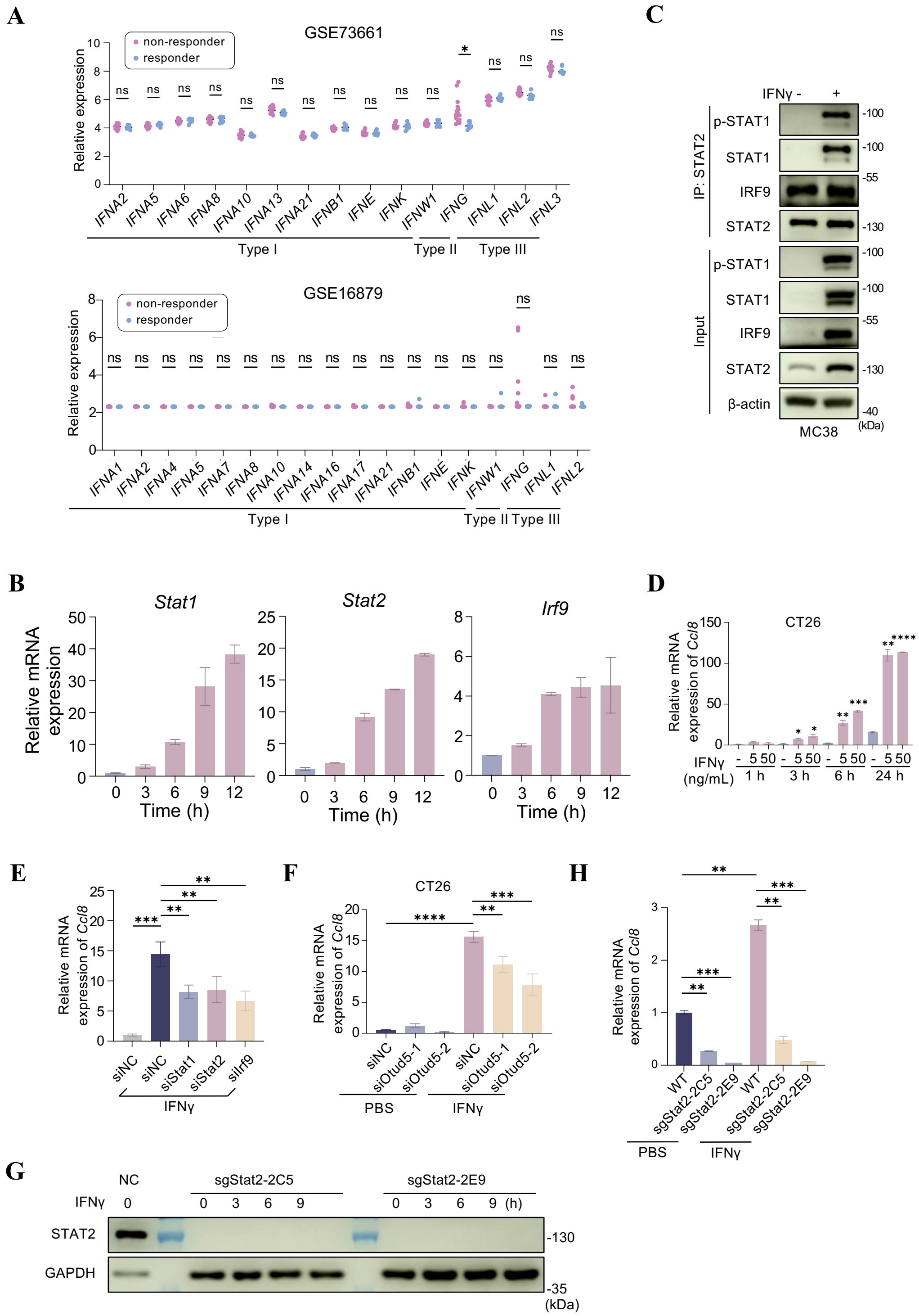

Figure S4. IFNγ activates *Ccl8* expression via ISGF3

(A) The relative expression level of interferons from datasets GSE73662 and GSE16879.

(B) qPCR analysis of *Stat1*, *Stat2* and *Irf9* expression in MC38 cells treated with IFNγ (50 ng/mL) for indicated time periods.

(C) Immunoprecipitation (IP) analysis of lysates from MC38 treated with or without IFNγ (50 ng/mL) for 12 hours.
(D) qPCR analysis of *Ccl8* expression in CT26 cells treated with PBS or IFNγ (5 or 50 ng/mL) for the indicated time periods.
(E) qPCR analysis of the relative mRNA expression of *Ccl8* in CT26 48 hours after siRNA transfection followed by treatment of IFNγ (50 ng/mL) for 6 hours.
(F) qPCR analysis of *Ccl8* expression in CT26 cells depleted of *Otud5* and treated with IFNγ (50 ng/mL) for 6 hours.
(G) Western blot validation of CRISPR-mediated knockout (KO) of *Stat2* in MC38 cells, compared with control (NC) cells. 2C5 and 2E9 indicate two different subclones.
(H) qPCR analysis of *Ccl8* expression in CRISPR-generated *Stat2*-knockout MC38 cells treated with IFNγ (50 ng/mL) for 4 hours. 2C5 and 2E9 indicate two different subclones shown in (G).
Data are presented as mean ± SD. *, $p < 0.05$; **, $p < 0.01$; ***, $p < 0.001$; ****, $p < 0.0001$.

Figure S5

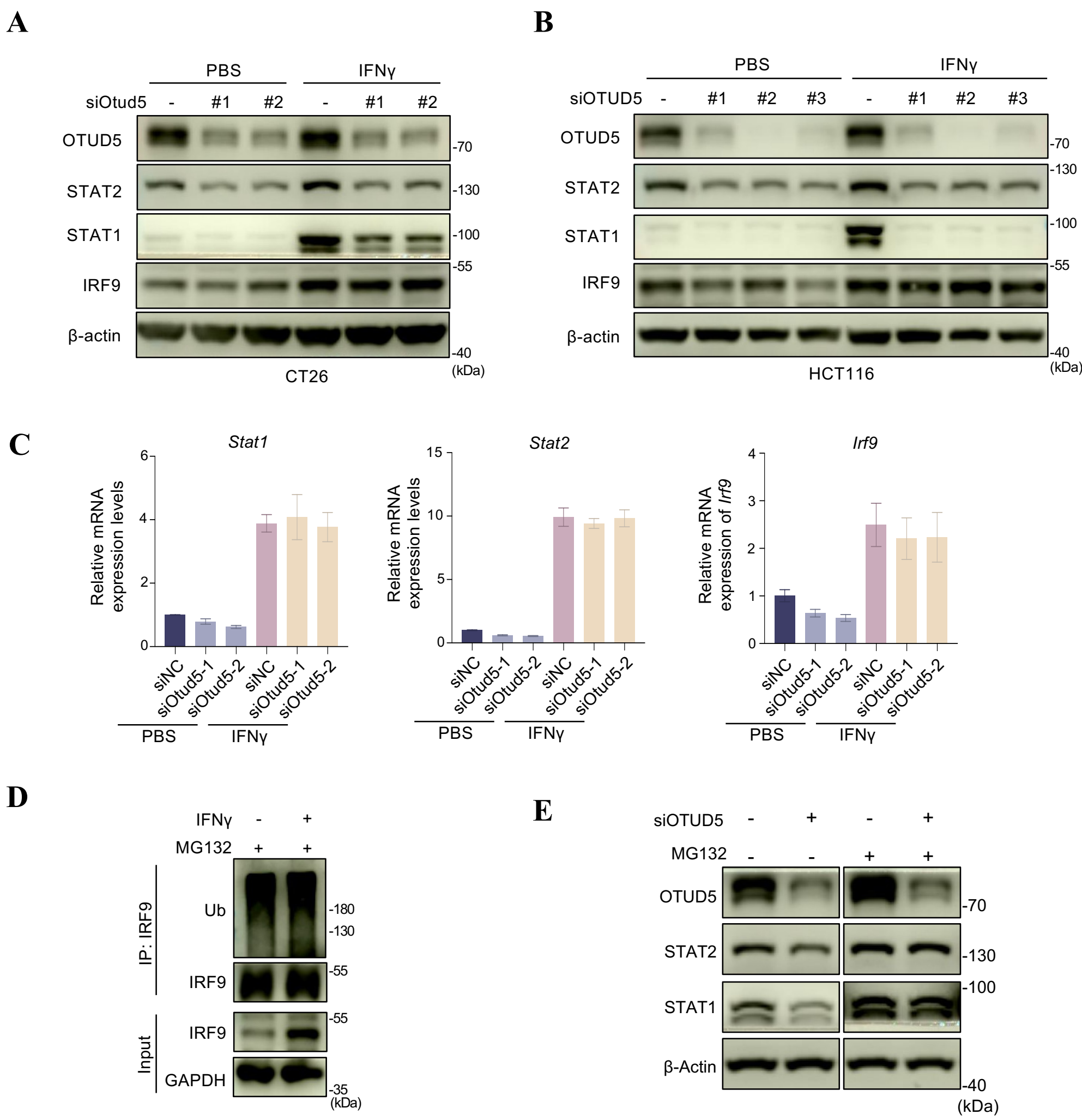

Figure S5. OTUD5 regulates STAT2 and STAT1 expression

(A) Western blot analysis 72 hours after siRNA transfection followed by treatment of IFNγ (50 ng/mL) for 6 hours in CT26.

(B) Western blot analysis 72 hours after siRNA transfection followed by treatment of IFNγ (50 ng/mL) for 6 hours in HCT116.

(C) qPCR analysis 72 hours after siRNA transfection followed by treatment of IFNγ (50 ng/mL) for 6 hours in MC38.

(D) Ubiquitination assay of IRF9. IRF9 was immunoprecipitated from MC38 cells treated with IFNγ (50 ng/mL) as well as MG132 (10 μM) for 16 hours.

(E) Western blot analysis of STAT2 and STAT1 in MC38 30 hours after siRNA transfection with or without MG132 (10 μM) followed by treatment of IFNγ (50 ng/mL) for 6 hours.

Data are presented as mean ± SD. *, $p < 0.05$; **, $p < 0.01$; ***, $p < 0.001$; ****, $p < 0.0001$.

Figure S6

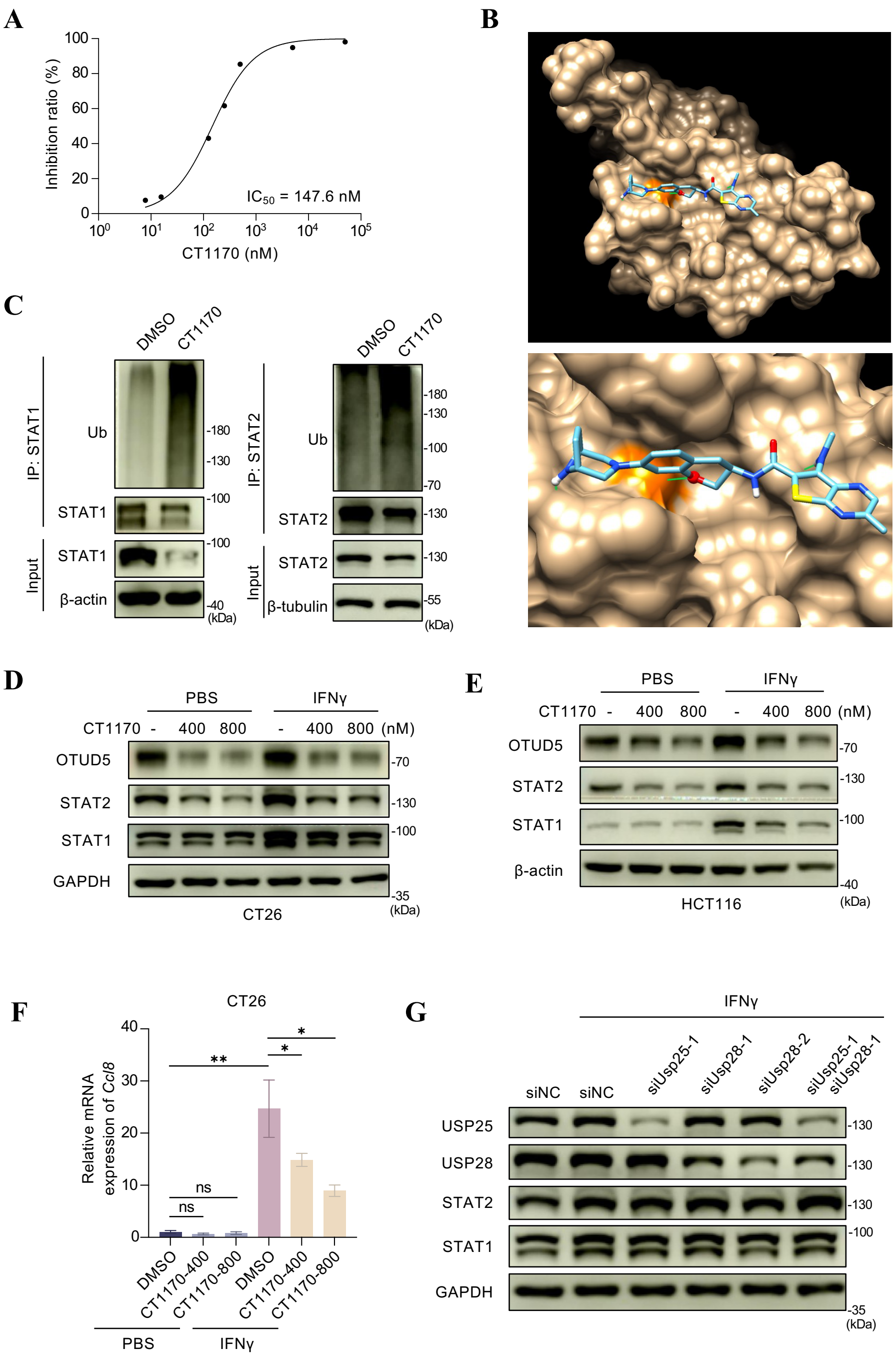

Figure S6. CT1170 is an inhibitor of OTUD5

(A) In vitro deubiquitination assay with Rhodamine110-ubquitin as a substrate. The inhibition by CT1170 was plotted to calculate IC_{50}.

(B) Molecular docking analysis of CT1170 and the catalytic domain of OTUD5 (3pfy).

(C) STAT2 and STAT1 ubiquitination assay. MC38 cells were pre-treated with DMSO or CT1170 (800 nM) for 2 hours followed by IFNγ (50 ng/mL) treatment for 6 hours.

(D) Western blot analysis of the effect of CT1170 in CT26. CT26 were treated with or without IFNγ (50 ng/mL) plus CT1170 (400 or 800 nM) for 6 hours.
(E) Western blot analysis of the effect of CT1170 in HCT116. HCT116 were treated with or without IFNγ (50 ng/mL) plus CT1170 (400 or 800 nM) for 6 hours.
(F) qPCR analysis of the effect of CT1170 on the relative mRNA expression of *Ccl8* in CT26. CT26 were treated with or without IFNγ (50 ng/mL) plus CT1170 (400 or 800 nM) for 24 hours.
(G) Western blot analysis in CT26 transfected with siRNA (siNC, siUsp25, siUsp28 or siUsp25 plus siUsp28) for 48 hours followed by treatment of PBS or IFNγ (50 ng/mL) for 6 hours.
Data are presented as mean ± SD. *, $p < 0.05$; **, $p < 0.01$; ***, $p < 0.001$; ****, $p < 0.0001$.

Figure S7

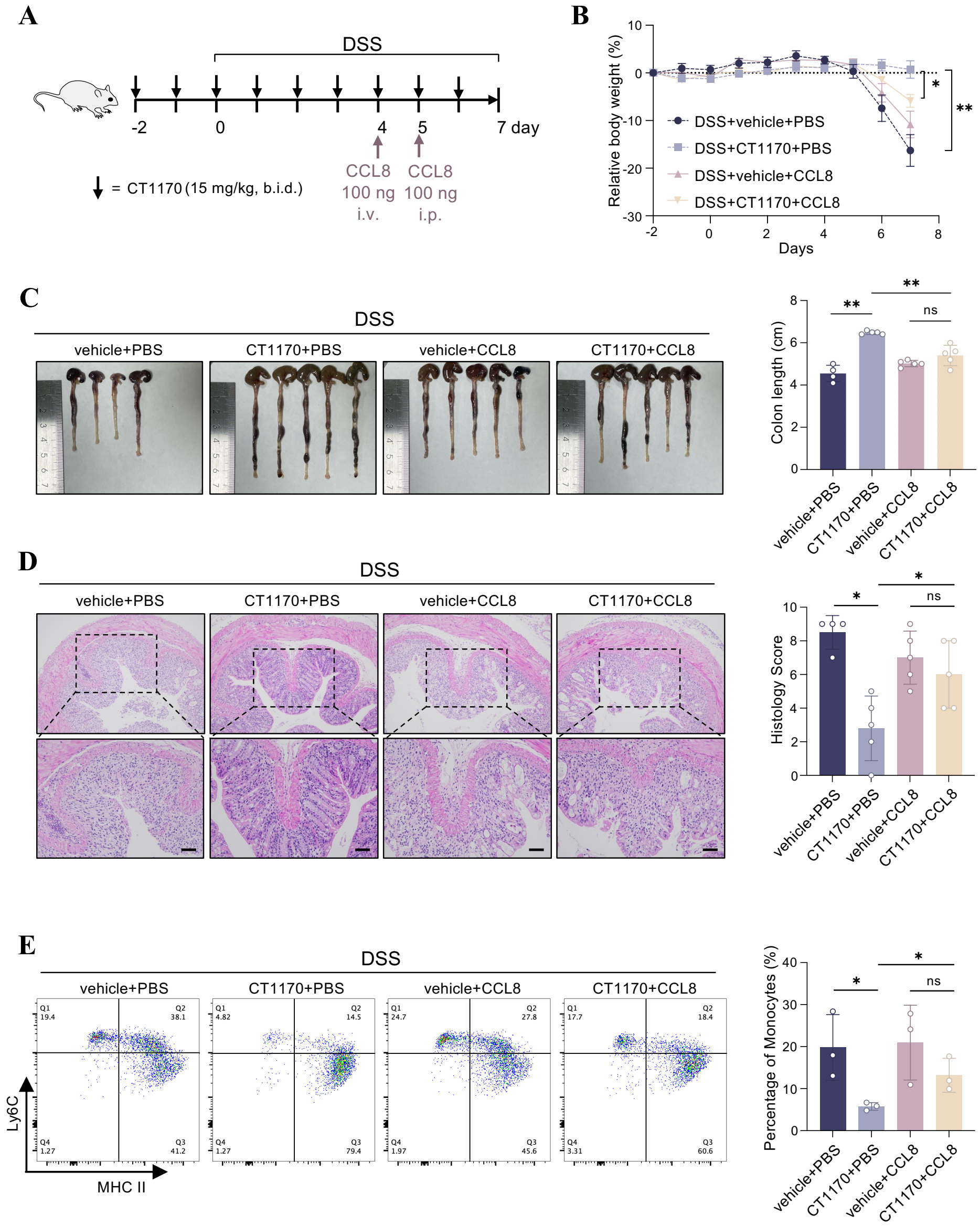

Figure S7. Exogenous CCL8 is capable of averting the therapeutic effect of OTUD5 inhibition.

(A) Schematic diagram of CT1170 and CCL8 administration in DSS-induced colitis model.

(B) Body weight measurements of the mice during the treatment (A) (n = 4 for DSS+vehicle+PBS group; n = 5 for the other three groups).

(C) The colons were isolated from the mice in different treatment groups on day 7 (n = 4 for DSS+vehicle+PBS group; n = 5 for the other three groups). The colon lengths were measured and presented.

(D) H&E staining and scoring of the colons in (C). Scale bar: 100 µm.

(E) Flow cytometric analysis of $Ly6C^{+}MHC\ II^{-}$ inflammatory monocytes in the lamina propria. Representative flow results and quantitation of the $Ly6C^{+}MHC\ II^{-}$ monocytes from all animals are shown.

Data are presented as mean ± SD (except in **B**, mean ± S.E.M. was presented). *, $p < 0.05$; **, $p < 0.01$; ***, $p < 0.001$; ****, $p < 0.0001$.
